# Tethered Blatter Radical for Molecular Grafting: Synthesis of 6-Hydroxyhexyloxy, Hydroxymethyl, and Bis(hydroxymethyl) Derivatives and Their Functionalization †

**DOI:** 10.3390/molecules27041176

**Published:** 2022-02-09

**Authors:** Szymon Kapuściński, Bindushree Anand, Paulina Bartos, Jose M. Garcia Fernandez, Piotr Kaszyński

**Affiliations:** 1 Faculty of Chemistry, University of Łódź, Tamka 12, 91-403 Łódź, Poland; szymkap@gmail.com (S.K.); paulina.bartos@chemia.uni.lodz.pl (P.B.); 2 Centre for Molecular and Macromolecular Studies, Polish Academy of Sciences, Sienkiewicza 112, 90-363 Łódź, Poland; bindushreearadhya93@gmail.com; 3 Institute for Chemical Research, CSIC, University of Sevilla, Americo Vespucio 49, 41092 Sevilla, Spain; 4 Department of Chemistry, Middle Tennessee State University, Murfreesboro, TN 37132, USA

**Keywords:** heterocycles, stable radicals, functional group transformation, azide–alkyne “click” reaction

## Abstract

Synthetic access to 7-CF_3_-1,4-dihydrobenzo[*e*][1,2,4]triazin-4-yl radicals containing 4-(6-hydroxyhexyloxy)phenyl, 4-hydroxymethylphenyl or 3,5-bis(hydroxymethyl)phenyl groups at the C(3) position and their conversion to tosylates and phosphates are described. The tosylates were used to obtain disulfides and an azide with good yields. The Blatter radical containing the azido group underwent a copper(I)-catalyzed azide–alkyne cycloaddition with phenylacetylene under mild conditions, giving the [1,2,3]triazole product in 84% yield. This indicates the suitability of the azido derivative for grafting Blatter radical onto other molecular objects via the CuAAC “click” reaction. The presented derivatives are promising for accessing surfaces and macromolecules spin-labeled with the Blatter radical.

## 1. Introduction

Functionalization of flat surfaces [1,2,3,4,5,6,7,8,9], polymers [10,11,12,13,14], well-defined macromolecules (dendrimers [15,16,17,18], cyclodextrins [19,20,21,22], fullerene [23], and nanotubes [24]), and nanoparticles [25,26,27,28,29,30] with stable radicals is becoming an important avenue for obtaining materials [31,32] for advanced technologies [33], which include organic electronics [11,34], spintronics [1,3,6], contrast agents in bioimaging [15,35,36], and energy storage [12,37,38,39]. This effort has concentrated mainly on the traditional stable radicals, such as nitroxides [6,14,15,16,17,18,19,32], tris(2,4,6,-trichlorophenyl)methyl (TTM) [7,9], and verdazyl [40], while the exploration of 1,4-dihydrobenzo[*e*][1,2,4]triazin-4-yls (so-called Blatter radicals) as active components of these materials began only recently and is rapidly intensifying.

Radicals have been chemisorbed onto an Au surface using the SR [1,2,6,26,41] and C≡CH [7,42] groups, and the latter was used to graft radicals onto reduced Si surfaces [42]. Chemisorption of radicals and typical organic molecules onto other surfaces has been accomplished using PO(OR)_2_ groups (metal oxide substrates, e.g., Fe_3_O_4_ [25] and LSMO [6]), COOH group (GaAs) [43] and siloxanes (for SiO_x_ and indium tin oxide–ITO–substrates) [44]. Grafting of radicals onto macromolecules has been achieved using a variety of acylation and condensation reactions [15,16,17,18,35,45,46]. One of the most efficient grafting methods involves the Cu(I)-catalyzed [3+2] cycloaddition (“click”) reaction between an azide and a terminal alkyne, leading to the formation of the [1,2,3]triazole ring with the 1,4-substitution pattern [47,48,49]. This approach has been applied to grafting ethynyl-containing radicals into systems with pending azido groups [24,25,29,50]. It should be added that radical polymers have also been obtained by polymerization of monomers containing stable radicals using, e.g., Rh catalysts, ring opening metathesis polymerization (ROMP), and electropolymerization methods [13,14,40].

**Figure 1:**
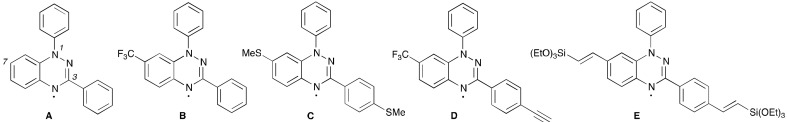
The structure of the prototypical Blatter radical (**A**), the “super stable” derivative (**B**), and functional derivatives (**C**–**E**).

The 1,4-dihydrobenzo[*e*][1,2,4]triazin-4-yls [33,51], formal derivatives of the prototypical 1,3-diphenyl derivative known as the Blatter radical [52] **A** (Figure 1), are exceptionally stable, π-delocalized radicals characterized by favorable redox behavior with a narrow electrochemical window (~1.2 V) [37,38] and a broad absorption in the visible range [53]. The stability of Blatter radical **A** is further enhanced by placing the CF_3_ group in the C(7) position leading to derivative **B**, the so-called “super stable” radical [54]. For these reasons, 1,4-dihydrobenzo[*e*][1,2,4]triazin-4-yls are promising paramagnetic structural elements of functional materials [33], and have been explored as photoconductive liquid crystals [55,56,57], sensors [58], photodetectors [59], and also in spintronics [41,60,61].

There are still relatively few studies on Blatter radicals chemisorbed on surfaces or grafted on macromolecules, mainly due to insufficiently developed access to derivatives with appropriate functional groups. For instance, Blatter radical containing two Au-anchoring SMe groups (**C**, Figure 1) was chemisorbed on the Au(111) surface and the interactions of the resulting molecular films with the surface were investigated in detail [41]. Acetylene derivative **D** was prepared and reacted with an azidonorbornene derivative under the “click” reaction conditions to give a norbornene-containing monomer, which was polymerized using the ROMP method. The acetylene derivative **D** could also be used for chemisorption onto the Au surface. Finally, bis(triethoxysilyl) derivative **E** was used for grafting the Blatter radical onto mesoporous silica [62] and could also be used for the functionalization of Si and metal surfaces with native oxides.

Despite progress in functional derivatives of the Blatter radical, there is a need to broaden the range of intermediates containing active functionalities for grafting onto diverse types of surfaces and macromolecules.

**Figure 2:**
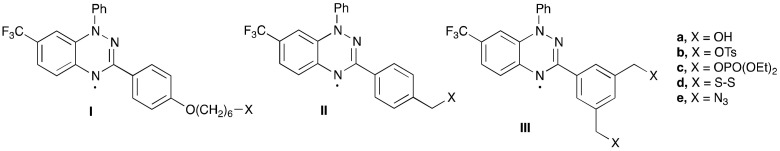
The structure of three types of radicals (**I**–**III**) for molecular grafting. Reported compounds: **Ia**–**Ie**, **IIa**–**IId**, **IIIa** and **IIIb**.

Herein we describe three derivatives of the “super stable” Blatter radical containing the 6-hydroxyhexyloxy (**Ia**, Figure 2) or hydroxymethyl (**IIa** and **IIIa**) substituents on the C(3)–Ph ring as key intermediates to functional derivatives suitable for grafting onto low dimensional systems. We report a conversion of the alcohols **Ia**–**IIIa** to the corresponding tosylates **Ib**–**IIIb** and phosphates **Ic** and **IIc**, and transformations of the tosylates to disulfides **Id** and **IId**, and azide **Ie**. As a proof of concept, we demonstrate the “click” reaction of azide **Ie** with an alkyne.

## 2. Results and Discussion

### 2.1. Synthesis of Hydroxyl Derivatives **Ia**–**IIIa**

**Scheme 1:**
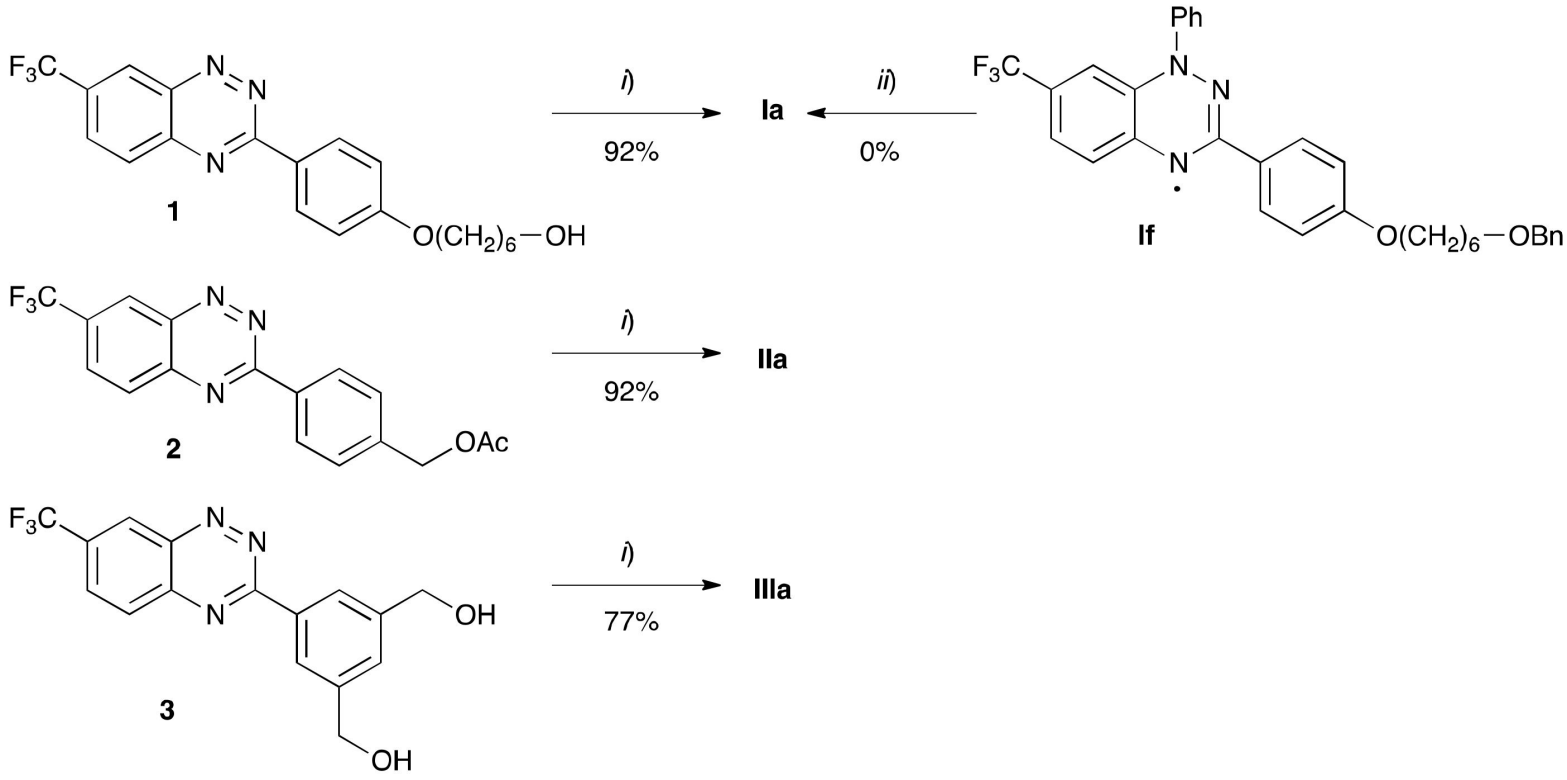
Synthesis of radicals (**Ia**–**IIIa**). *Reagents and conditions*: (*i*) 1. PhLi, THF, Ar, −5 °C, 1 h; 2. Air, 0.5 h, 77–92% yield; (*ii*) 1. Pd/C, H_2_, 50 psi, THF/EtOH, 2 days, 2. Air, 0% yield.

**Scheme 2:**
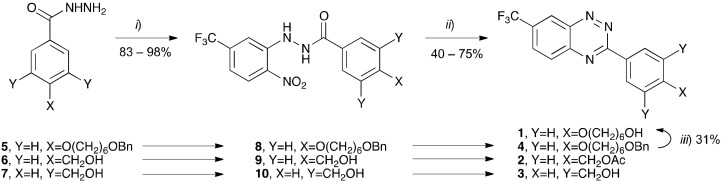
Preparation of benzo[*e*][1,2,4]triazines 1–3. *Reagents and conditions*: (*i*) 1-fluoro-2-nitro-5-trifluoromethylbenzene, DMSO, 70 °C, 7 h; (*ii*) 1. Sn, glacial AcOH, rt for 2 h, then 115 °C for 0.5 h; 2. NaIO_4_; (*iii*) 1. Pd/C, H_2_, THF/EtOH, 2 days; 2. Air.

Radicals **Ia**–**IIIa** containing versatile hydroxyl groups were prepared using the recently discovered regioselective azaphilic addition of aryllithium to benzo[*e*][1,2,4]triazines [63]. Thus, phenyllithium was reacted with benzo[*e*][1,2,4]triazines **1**–**3**, and the resulting anions were oxidized with air to the corresponding radicals **Ia**–**IIIa**, which were conveniently isolated by column chromatography (SiO_2_ support) in yields up to 92% (Scheme 1). It should be noted that both hydroxy (**1** and **3**) and acetoxy (**2**) derivatives were suitable starting materials for this reaction affording the corresponding radicals in comparable yields. The route to **Ia** involving addition of phenyllithium to compound **4**, the *O*-benzyl protected alcohol **1** (Scheme 2), followed by Pd-catalyzed reductive debenzylation of the resulting radical **If** (Scheme 1) turned out to be inefficient. While the PhLi addition and formation of **If** worked well (yield up to 64%, Scheme 2), debenzylation of **If** gave only decomposition products. This presumably resulted from the more vigorous conditions needed for the removal of the *O*-benzyl group in the alkyl benzyl ethers than in the aryl analogues. 

The requisite alcohols **1** and **3** and acetate **2** were obtained in two steps following an established general procedure [57,63,64], as shown in Scheme 2. Thus, the readily available benzhydrazides **5**–**7** were N-arylated with 1-fluoro-2-nitro-5-trifluoromethylbenzene. The resulting hydrazides **8**–**10** were subsequently cyclized under reductive conditions (Sn powder/AcOH) followed by oxidation of the dihydro products with Ag_2_O or NaIO_4_ giving benzo[*e*][1,2,4]triazines **2**–**4** in good overall yields (40–75%). Interestingly, during reductive cyclization of **9** at temperatures above 100 °C, the hydroxymethyl group underwent esterification with AcOH, used as the solvent and reagent in this reaction, and acetate **2** was isolated in 45% yield. The analogous acetate was not observed in the case of cyclization of hydrazide **10** conducted at ambient temperatures.

The hydroxyhexyloxy derivative **1** was obtained in 31% overall yield by Pd-catalyzed debenzylation of **4**, followed by aerial oxidation of the dihydro form. As noted above, debenzylation of **4** required longer-than-typical reaction times. The obtained hydroxy derivative **1** turned out to be sensitive to elevated temperatures: concentration of solutions of purified **1** on a rotavap at 40 °C resulted in its decomposition and formation of a foul-smelling orange oil. Handling of **1** at lower temperatures avoided this problem, and pure product was obtained.

Benzhydrazides **5**–**7** were obtained by hydrazinolysis of the corresponding methyl benzoates with hydrazine hydrate.

### 2.2. Synthesis of Tosylates and Phosphates

**Scheme 3:**
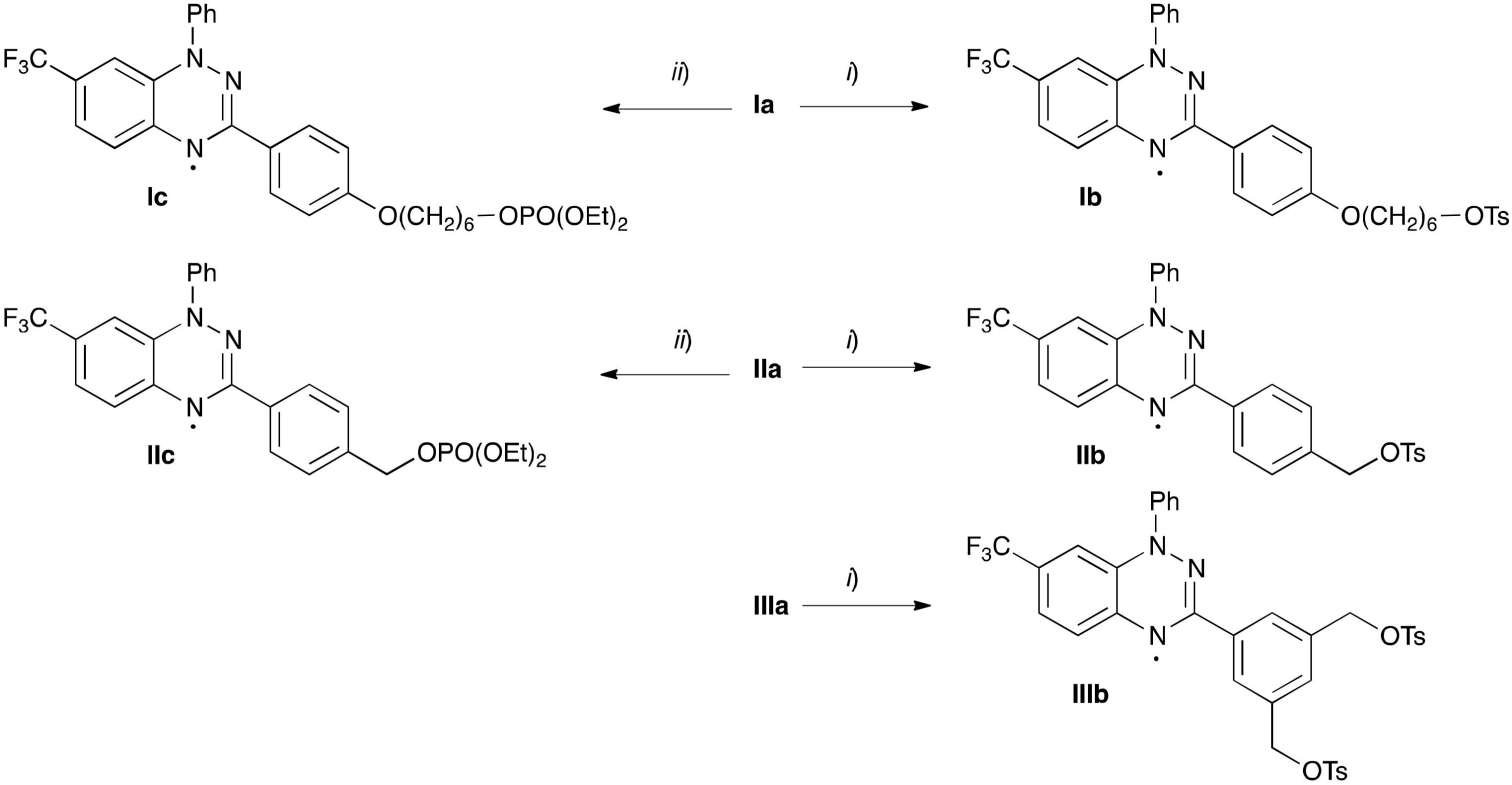
Synthesis of tosylates **Ib**–**IIIb** and phosphates **Ic** and **IIc**. *Reagents and conditions*: (*i*) TsCl, pyridine, CH_2_Cl_2_, rt, Ar, overnight, 82–96% yield; (*ii*) (EtO)_2_POCl, DMAP, Et_3_N, THF, rt, 1 h, Ar, 83–86% yield.

Reactions of alcohols **Ia**–**IIIa** with tosyl chloride gave the desired tosylates **Ib**–**IIIb** in 82–96% yield (Scheme 3). The relatively high stability of **Ib** allowed for isolation of the pure compound using standard silica gel chromatography. In contrast, tosylates **IIb** and **IIIb** were sensitive to chromatography conditions and were used for the next step as crude materials.

Phosphorylation of alcohols **Ia** and **IIa** with diethyl chlorophosphate in the presence of DMAP and Et_3_N gave the phosphates **Ic** and **IIc**, respectively, isolated in about 85% yield (Scheme 3).

### 2.3. Transformation of Tosylates: Preparation of Disulfides and Azide

**Scheme 4:**
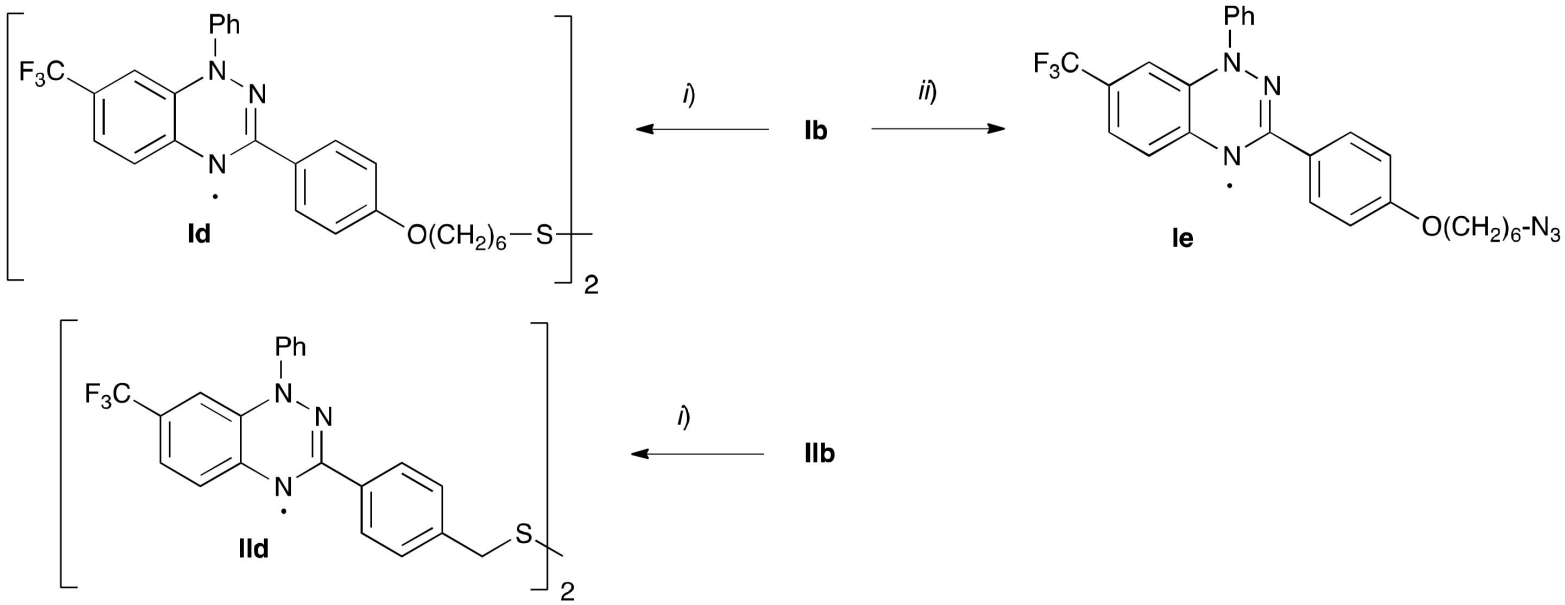
Synthesis of disulfides **Id** and **IId**, and azide **Ie**. *Reagents and conditions*: (*i*) 1. Na_2_S_2_O_3_, DMSO, 60 °C, overnight, 2. I_2_, CH_2_Cl_2_, rt, 10 min, 18% yield; (*ii*) NaN_3_, DMF, 50 °C, Ar, 5 h, 42–73% yield.

Disulfides **Id** and **IId** were obtained in 18% yield from tosylates **Ib** and **IIb** using a general procedure [65] involving reactions with the thiosulfate (S_2_O_3_^2−^) nucleophile in DMSO, followed by oxidation of the resulting mercaptan with I_2_ (Scheme 4).

In contrast, the preparation of azide **Ie** was straightforward and more efficient. Thus, reaction of toslyate **Ib** with NaN_3_ in DMF gave azide **Ie** isolated in yields up to 73% yield (Scheme 4).

### 2.4. Copper(I)-Catalyzed Azide-Alkyne Cycloaddition of Azide **Ie**

**Scheme 5:**
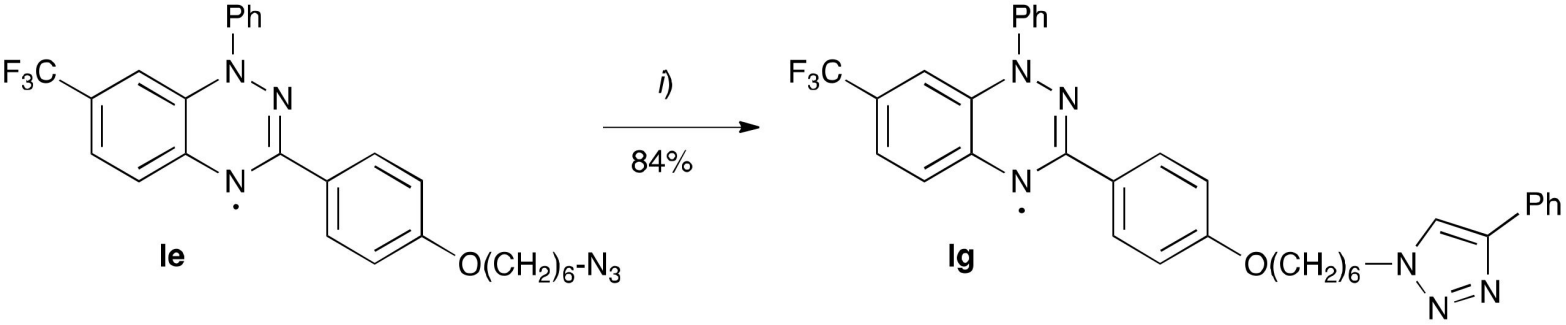
Synthesis of **Ig** via azide-alkyne “click” reaction. *Reagents and conditions:* (*i*) Phenylacetylene, CuSO_4_·5H_2_O, sodium *L*-ascorbate, benzoic acid, *t*-BuOH/H_2_O 1:2, rt, 5 h, 84% yield.

Compound **Ie** represents the first azido derivative of the Blatter radical, and its suitability for the Cu(I)-catalyzed cycloaddition reaction (“click”) with alkynes, the CuAAC reaction, required experimental verification. Thus, the azide **Ie** was reacted with phenylacetylene in the presence of Cu(I), generated in situ from CuSO_4_ and sodium ascorbate, according to a general literature method [66]. The “click” product, [1,2,3]triazole **Ig**, was isolated in a high yield of 84% (Scheme 5). This result compares to 53% yield of [1,2,3]triazole formation in an analogous CuAAC reaction of acetylene-substituted Blatter radical **D** (Figure 1) with an azido derivative of norbornene [13].

Pure **Ig** showed no decomposition during storage for 4 years under ambient conditions, according to thin-layer chromatography analysis.

### 2.5. Spectroscopic Characterization of Radicals

**Figure 3:**
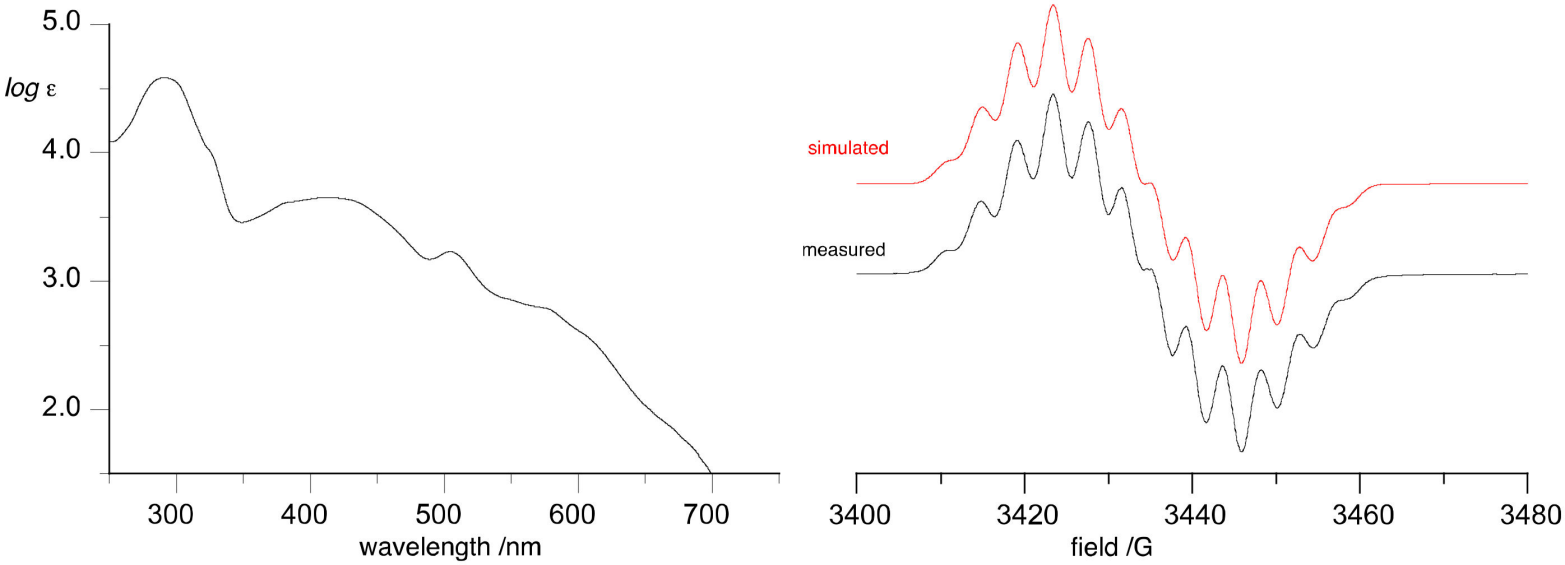
Electronic absorption spectrum in CH_2_Cl_2_ (**left**) and ERP spectrum in benzene (**right**) for **Ia**.

All radicals **I**–**III** exhibit low-intensity broad absorption in the entire visible range, as shown for **Ia** in Figure 3. Consequently, the compounds appear dark brown in solutions and nearly black in the solid state. EPR analysis of the radicals conducted in benzene solutions revealed seven principal lines resulting from hyperfine splitting with three ^14^N nuclei modulated with additional smaller splitting by ^19^F and ^1^H nuclei, as shown for derivative **Ia** in Figure 3. For some radicals, the principal lines are less resolved, presumably due to aggregation in benzene solutions (see the Supplementary Materials). Analysis demonstrated that the *a*_N_ *hfcc* values for radicals **I**–**III** are consistent with those for other Blatter radical derivatives, and are about 7.6 G for *a*_N(1)_ and about 4.5–4.9 G for *a*_N(2)_ and *a*_N(4)_. Simulation of the experimental spectra indicates that the *a*_F_ *hfcc* value is in a range of 3.2–3.6 G.

## 3. Conclusions

The “super stable” Blatter radical **B** was substituted at the C(3) phenyl ring with a long tether (O(CH_2_)_6_-X, **I**), a short tether (CH_2_-X, **II**) or two anchoring groups (2 × CH_2_-X, **III**). The key intermediates contain the hydroxyl group (X = OH, **a**), which could be used for grafting in condensation (acylation) and addition (e.g., carbamination) reactions. The hydroxyl derivatives **Ia**–**IIIa** are efficiently converted to tosylates **Ib**–**IIIb**, which served as electrophilic intermediates to disulfides **Id** and **IId** (for chemisorption onto Au surfaces) and azide **Ie** (for the CuAAC reaction). Tosylates **IIb** and **IIIb** can additionally be exploited to attach the Blatter radical to hydroxyl-functionalized partners through the formation of benzyl-type ether linkages. Following prior work on the synthesis and supramolecular properties of cyclodextrin–xylylene hybrids [67,68,69,70], we have conducted preliminary experiments supporting the viability of such an approach, and the results will be published in due course.

The demonstration of efficient “click” reaction of azide **Ie** with PhC≡CH paves the way to grafting radical **B** onto surfaces and macromolecules functionalized with terminal ethynyl groups. This method represents a more versatile approach to paramagnetic materials, since many macromolecules substituted with the propargyl group are available.

The presented results constitute a promising approach to novel paramagnetic polymers with high radical density, e.g., for polymer electrodes with high charge storage capacity and for high-density paramagnetic surfaces for spintronic applications. This work is continued in our laboratory.

## 4. Materials and Methods

**General.** Reagents and solvents were purchased and used as received. THF was dried over Na metal in the presence of benzophenone and distilled before use. Column chromatography was performed on silica gel. For separation of radicals silica gel was passivated by mixing with CH_2_Cl_2_ containing 2% of Et_3_N and removal of the solvent to dryness (rotovap). Reported yields refer to analytically pure samples. NMR spectra were recorded with a Bruker AVIII 500 or 600 instrument. Chemical shifts are reported relative to solvent (CDCl_3_) residual peaks (^1^H NMR: *δ* = 7.26 ppm and ^13^C NMR: *δ* = 77.16 ppm) [71]. All ^13^C NMR spectra are proton-decoupled. IR spectra were measured in KBr pellets with a FTIR NEXUS spectrometer. High-resolution mass spectrometry (HRMS) measurements were performed using SYNAPT G2-Si High-Definition Mass Spectrometry equipped with an ESI or APCI source and Quantitative Time-of-Flight (QuanTof) mass analyzer. Melting points were determined in capillaries with a MEL-TEMP II apparatus and are uncorrected. If not stated otherwise, reactions were carried out under argon atmosphere in flame-dried flasks with addition of the reactants via syringe. Subsequent manipulations were conducted in air.

EPR spectra of radicals **I**–**III** were recorded on an X-band EMX-Nano EPR spectrometer at ambient temperature using dilute and degassed solutions in distilled benzene in a concentration range of 2–5 × 10^−4^ M. Additional details are shown in the SI.

**Preparation of radicals Ia–IIIa via PhLi addition to benzo[*e*][1,2,4]triazines 1-3. A general method.** To a solution of the appropriate benzo[*e*][1,2,4]triazine derivative **1**, **2**, or **3** (0.792 mmol) in dry THF (10 mL), PhLi (1.9 M in dibutyl ether, 1.24 mL, 2.360 mmol) was added dropwise at −5 °C under argon and the reaction mixture was stirred at this temperature for 1 h, then for 1 h at rt under air. Water was added and the product was extracted with CH_2_Cl_2_ (3×). The combined organic extracts were dried (Na_2_SO_4_), and volatiles were removed on a rotavap. Pure product was isolated by column chromatography followed by recrystallization (MeCN).

**1-Phenyl-3-[4-(6-hydroxyhexyloxy)phenyl]-7-trifluoromethyl-1,4-dihydrobenzo[*e*][1,2,4]triazin-4-yl (Ia)**. Starting from triazine **1**. Yield: 322 mg (87–94% range yield) of pure **Ia** as a brown solid: mp 145–146 °C; IR (KBr) *v* 3400, 2932, 2854, 1607, 1514, 1393, 1356, 1314, 1246, 1118, 1063, 841 cm^−1^; UV (CH_2_Cl_2_) λ_max_ (log ε) 291 (4.58), 413 (3.65), 504 (3.20) nm; EPR (benzene) *a_N_* 7.57, 4.54, 4.87, *a_F_* 3.51 G, *g* = 2.0048; HRMS (ESI-TOF) [M+H]^+^ *m*/*z* calcd for C_26_H_26_F_3_N_3_O_2_: 469.1977; found: 469.1984. Anal. Calcd for C_26_H_25_F_3_N_3_O_2_: C, 66.66; H, 5.38; N, 8.97. Found: C, 66.46; H, 5.45; N, 8.77.

**1-Phenyl-3-(4-hydroxymethylphenyl)-7-trifluoromethyl-1,4-dihydrobenzo[*e*][1,2,4]triazin-4-yl (IIa)**. Staring from triazine **2**. The crude product was passed through a passivated silica gel plug (pet. ether/AcOEt 1:1) and recrystallized (MeCN) to give 279 mg (92% yield) of pure **IIa** as an olive solid: mp 204–205 °C; IR (KBr) *v* 3366, 3275, 3054, 1592, 1489, 1397, 1355, 1315, 1265, 1166, 1117, 1062, 1013, 905 cm^−1^; EPR (benzene) *a_N_* 7.40, 4.59, 4.90, *a_F_* 3.24 G, *g* = 2.0034; HRMS (ESI-TOF) [M+H]^+^ *m*/*z* calcd for C_21_H_16_F_3_N_3_O: 383.1245; found: 383.1233. Anal. Calcd for C_21_H_15_F_3_N_3_O: C, 65.97; H, 3.95; N, 10.99. Found: C, 66.10; H, 4.06; N, 11.25.

**1-Phenyl-3-[3,5-bis(hydroxymethyl)phenyl]-7-trifluoromethyl-1,4-dihydrobenzo[*e*][1,2,4]triazin-4-yl (IIIa).** Starting from triazine **3**. The crude product was passed through a passivated silica gel plug (pet. ether/AcOEt 1:1) and recrystallized (MeCN) to give **IIIa** as an olive solid (251 mg, 77% yield): mp 180–181 °C; IR (KBr) *v* 3394, 3219, 3066, 2858, 1709, 1592, 1490, 1427, 1396, 1322, 1278, 1130, 1070, 875, 841, 765, 692 cm^−1^; EPR (benzene) *a_N_* 7.64, 4.60, 4.79, *a_F_* 3.49 G, *g* = 2.0038; HRMS (ESI-TOF) [M+H]^+^ *m*/*z* calcd for C_22_H_18_F_3_N_3_O_2_: 413,1351; found: 413.1355.

**Preparation of 1-phenyl-3-[4-(6-tosyloxyhexyloxy)phenyl]-7-trifluoromethyl- 1,4-dihydrobenzo[*e*][1,2,4]triazin-4-yl (Ib)**. To a solution of alcohol **Ia** (500 mg, 1.07 mmol) in dry CH_2_Cl_2_ (6 mL), pyridine (0.25 mL, 3.10 mmol) and tosyl chloride (305 mg, 1.60 mmol) were added and the reaction mixture was stirred at rt overnight under argon. The reaction mixture was washed with H_2_O, brine and dried (Na_2_SO_4_). Organic solvents were removed on a rotavap and the residue was passed through a silica gel plug (CH_2_Cl_2_/EtOAc 19:1) to give 637 mg (96% yield) of pure tosylate **Ib** as a brown solid: mp 120–121 °C; IR (KBr) *v* 2940, 2864, 1605, 1390, 1355, 1316, 1244, 1172, 1119, 960, 842 cm^−1^; HRMS (ESI-TOF) [M+H]^+^ *m*/*z* calcd for C_33_H_32_F_3_N_3_O_4_S: 623.2066; found: 623.2062. Anal. Calcd for C_33_H_31_F_3_N_3_O_4_S: C, 63.65; H, 5.02; N, 6.75; S, 5.15. Found: C, 63.65; H, 5.16; N, 6.63; S, 5.03.

**Preparation of tosylates IIb and IIIb. A general method.** To a solution of alcohol **IIa** or **IIIa** (0.79 mmol) and tosyl chloride (300 mg, 1.57 mmol) in dry THF (10 mL), 60% NaH in mineral oil (475 mg, 11.85 mmol) was added in portions over 30 min. The suspension was stirred at rt for another 30 min, and water was added dropwise to neutralize the unreacted NaH. The reaction mixture was extracted with CH_2_Cl_2_ (3×). The combined organic extracts were dried (Na_2_SO_4_) and concentrated in vacuo. The crude product was passed through a silica gel plug passivated with Et_3_N (pet ether/AcOEt 4:1) to give **IIb** or **IIIb** as green-brown solid. The product was immediately used for the next step without further purification

**1-Phenyl-3-[4-(tosyloxymethyl)phenyl]-7-trifluoromethyl-1,4-dihydrobenzo[*e*][1,2,4]triazin-4-yl (IIb).** Starting from **IIa**. Yield: 413 mg (98% yield) of **IIb** as a brown solid; HRMS (ESI-TOF) [M+H]^+^ *m*/*z* calcd for C_28_H_22_F_3_N_3_O_3_S: 537.1334; found: 537.1330.

**1-Phenyl-3-[3,5-bis(tosyloxymethyl)phenyl]-7-trifluoromethyl-1,4-dihydrobenzo[*e*][1,2,4]triazin-4-yl (IIIb).** Starting from **IIIa**. Yield: 558 mg (98% yield) of **IIIb** as a brown solid; HRMS (ESI-TOF) [M+H]^+^ HRMS, *m*/*z* calcd for C_36_H_30_F_3_N_3_O_6_S_2_: 721,1528; found: 721.1536.

**Preparation of phosphates Ic and IIc. A general method**. To a solution of alcohol **Ia** or **IIa** (0.2 mmol, 1.0 equiv.), DMAP (0.02 mmol, 0.1 equiv.) and Et_3_N (1.0 mmol, 5 equiv.) dissolved in THF (2 mL) diethyl chlorophosphate (1.0 mmol, 5 equiv.) was added slowly via syringe. During the addition, a white precipitate formed. The reaction mixture was stirred at rt for 1 h until substrate was no longer present in the reaction mixture (TLC control). The reaction was quenched with sat. NH_4_Cl solution, extracted with CH_2_Cl_2_, and the product was purified by column chromatography (pet. ether/AcOEt 3:2).

**1-Phenyl-3-[4-(6-diethoxyphosphoryloxyhexyloxy)phenyl]-7-trifluoromethyl- 1,4-dihydrobenzo[*e*][1,2,4]triazin-4-yl (Ic).** Starting from **Ia**. Yield: 103.9 mg (86% yield) of phosphate **Ic** as a soft waxy material; IR (KBr) *v* 2925, 2854, 1607, 1490, 1399, 1318, 1248, 1165, 1108, 1027, 829, 698 cm^−1^; EPR (benzene) *a_N_* 7.56, 4.78, 4.53, *a_F_* 3.56 G, *g* = 2.0037; HRMS (ESI-TOF) [M+H]^+^ *m*/*z* calcd for C_30_H_35_N_3_O_5_F_3_P: 605.2266; found: 605.2273.

**1-Phenyl-3-[4-(diethoxyphosphoryloxymethyl)phenyl]-7-trifluoromethyl-1,4-dihydrobenzo[*e*][1,2,4]triazin-4-yl (IIc).** Starting from **IIa**. Yield: 86.1 mg (83% yield) of phosphate **IIc** as a soft waxy material; IR (KBr) *v* 2925, 2854, 1593, 1492, 1422, 1323, 1265, 1166, 1120, 1036, 824, 698 cm^−1^; EPR (benzene) *a_N_* 7.59, 4.63, 4.72, *a_F_* 3.56 G *g* = 2.0046; HRMS (ESI-TOF) [M+H]^+^ *m*/*z* calcd for C_25_H_25_N_3_O_4_F_3_P: 519.1535; found: 519.1529.

**Preparation of disulfides Id and IId. A general method**. A mixture of tosylate **Ib** or **IIb** (0.64 mmol) and solid Na_2_S_2_O_3_ (102 mg, 0.64 mmol) in DMSO (15 mL) was stirred at 60 °C overnight. The reaction mixture was cooled to rt and extracted with AcOEt (3×). The combined organic extracts were dried (Na_2_SO_4_) and concentrated in vacuo. The residue was dissolved in CH_2_Cl_2_ (10 mL) and solid I_2_ (49 mg, 0.19 mmol) was added and stirred at rt for 10 min, filtered, the solid was washed with CH_2_Cl_2_ and the filtrate was evaporated. The crude product was passed through a passivated silica gel plug (pet. ether/AcOEt 5:1) and recrystallized (MeCN) to give pure product **Id** or **IId** as brown solids.

**Disulfide Id.** Starting from **Ib**. Yield: 55.0 mg (18% yield); mp 73 °C; IR (KBr) *v* 2936, 2855, 1605, 1512, 1487, 1426, 1398, 1319, 1251, 1170, 1115, 1060, 840, 695 cm^−1^; UV (CH_2_Cl_2_) λ_max_ (log ε) 292 (4.82), 384 (4.01), 503 (3.34) nm; EPR (benzene) *a_N_* 7.32, 4.66, 4.55, *a_F_* 3.32 G, *g* = 2.0038; HRMS (ESI-TOF) [M]^+^ *m*/*z* calcd for C_52_H_48_F_6_N_6_O_2_S_2_: 966.3184; found: 966.3218. Anal. Calcd for C_52_H_48_F_6_N_6_O_2_S_2_: C, 64.58; H, 5.00; N, 8.69; S, 6.63. Found: C, 64.30; H, 5.10; N, 8.63; S, 6.84.

**Disulfide IId.** Starting from **IIb**. Yield: 45.8 mg (18% yield); mp 170 °C; IR (KBr) *v* 3073, 1592, 1488, 1397, 1316, 1264, 1116, 1062, 905, 844, 763, 693 cm^−1^; UV (CH_2_Cl_2_) λ_max_ (log ε) 284 (4.89), 374 (4.04), 430 (3.78), 498 (3.42) nm; HRMS (ESI-TOF) [M]^+^ *m*/*z* calcd for C_42_H_28_F_6_N_6_S_2_: 794.1721; found: 794.1726. Anal. Calcd for C_42_H_28_F_6_N_6_S_2_: C, 63.47; H, 3.55; N, 10.57; S, 8.07. Found: C, 63.45; H, 3.61; N, 10.46; S, 8.05. 

**Preparation of 1-phenyl-3-[4-(6-azidohexyloxy)phenyl]-7-trifluoromethyl-1,4-dihydrobenzo[*e*][1,2,4]triazin-4-yl (Ie).** To a solution of tosylate **Ib** (750 mg, 1.204 mmol) in dry DMF (18 mL) sodium azide (391 mg, 6.02 mmol) was added in one portion and the reaction mixture was stirred for 5 h at 50 °C under argon. After the reaction is completed (no starting material detected by TLC), H_2_O and brine were added, and the product was extracted with CH_2_Cl_2_ (3×). The organic layer was washed with H_2_O (3×), then with brine, and dried (Na_2_SO_4_). Solvents were removed and the residue was purified by column chromatography (SiO_2_, CH_2_Cl_2_ 100%) to give 432 mg (73% yield) of pure azide **Ie** as brown solid: mp 88-90 °C; IR (KBr) *v* 2939, 2857, 2098 (N_3_), 1606, 1397, 1354, 1317, 1247, 1113 cm^−1^; EPR (benzene) *a_N_* 7.57, 4.82, 4.98, *a_F_* 3.33 G, *g* = 2.0049; HRMS (ESI-TOF) [M+H]^+^ *m*/*z* calcd for C_26_H_25_F_3_N_6_O: 494.2042; found: 494.2031.

**Preparation of 1-phenyl-3-[4-(6-benzyloxyhexyloxy)phenyl]-7-trifluoromethyl -1,4-dihydrobenzo[*e*][1,2,4]triazin-4-yl (If)**. Radical **If** was obtained in 44–64% yield by addition of PhLi to triazine **4** according to the general procedure for preparation of alcohol **Ia**–**IIIa**. The crude product was passed through a silica gel plug (pet. ether/Et_2_O 3:2) and recrystallized (EtOH) to give pure **If** as a brown solid: mp 98–100 °C; HRMS (ESI-TOF) [M]^+^ *m*/*z* calcd for C_33_H_31_F_3_N_3_O_2_: 558.2368; found: 558.2363. Anal. Calcd for C_33_H_31_F_3_N_3_O_2_: C, 70.95; H, 5.59; N, 7.52. Found: C, 70.95; H, 5.56; N, 7.62.

**Preparation of 1-phenyl-3-[4-(6-[1,2,3]triazolylhexyloxy)phenyl]-7-trifluoromethyl-1,4-dihydrobenzo[*e*][1,2,4]triazin-4-yl (Ig)**. To a solution of CuSO_4_ × 5H_2_O (~3 mg), sodium ascorbate (~3 mg) and benzoic acid (~3 mg) in *t*-BuOH/H_2_O 1:2 (4 mL) a mixture of phenylacetylene (20.0 mg, 0.196 mmol) and azide **Ie** (95.0 mg, 0.192 mmol) was added at rt. The resulting mixture was stirred for 5 h (TLC monitoring, CH_2_Cl_2_, R_f_ = 0.08 and 0.45 for product **Ig** and azide **Ie**, respectively). CH_2_Cl_2_ was added and the organic phase was washed with H_2_O and brine and dried (Na_2_SO_4_). Solvents were removed in vacuum and the product was purified by column chromatography (SiO_2_, CH_2_Cl_2_ 100% gradient to CH_2_Cl_2_/EtOAc 4:1) to give 97 mg (84% yield) of pure triazole **Ig** as a brown solid: mp 160 °C dec.; IR (KBr) *v* 2943, 1607, 1400, 1357, 1319, 1246, 1121, 837, cm^−1^; UV (CH_2_Cl_2_) λ_max_ (log ε) 290 (4.58), 411 (3.62), 504 (3.20) nm; EPR (benzene) *a_N_* 7.59, 4.73, 4.87, *a_F_* 3.50 G, *g* = 2.0054; HRMS (ESI-TOF) [M+H]^+^ *m*/*z* calcd for C_34_H_31_F_3_N_6_O: 596.2511; found: 596.2506. Anal. Calcd for C_34_H_30_F_3_N_6_O: C, 68.56; H, 5.08; N, 14.11. Found: C, 68.59; H, 5.00; N, 13.97.

**Preparation of 3-[4-(6-hydroxyhexyloxy)phenyl]-7-trifluoromethylbenzo[*e*][1,2,4] triazine (1).** A solution of benzyloxy derivative **4** (3.00 g 6.23 mmol) in THF (35 mL) was added to a suspension of 5% Pd/C (2.60 g) in EtOH (35 mL), and the resulting mixture was hydrogenated (50 psi) overnight. The reaction mixture was passed through a Celite pad and oxidized by exposure to air (TLC monitoring) and the solvents were removed under reduced pressure (cold bath!). Crude product was purified by column chromatography (SiO_2_, CH_2_Cl_2_/EtOAc 4:1) to give 0.76 g (31% yield) of pure product **1** as a yellow solid: mp 144–145 °C; ^1^H NMR (600 MHz, CDCl_3_) δ 8.82 (s, 1H), 8.74 (d, *J* = 8.9 Hz, 2H), 8.16 (d, *J* = 8.8 Hz, 1H), 8.08 (dd, *J*_1_ = 8.9 Hz, *J*_2_ = 1.9 Hz, 1H), 7.10 (d, *J* = 8.9 Hz, 2H), 4.11 (t, *J* = 6.5 Hz, 2H), 3.69 (t, *J* = 6.5 Hz, 2H), 1.87 (quint, *J* = 7.0 Hz, 2H), 1.64 (quint, *J* = 7.1 Hz, 2H), 1.56 (quint, *J* = 7.5 Hz, 2H), 1.48 (quint, *J* = 7.4 Hz, 2H), 1.25 (s, 1H); ^13^C{^1^H} NMR (126 MHz, CDCl_3_) δ 162.8, 160.9, 144.9, 142.3, 131.2 (q, *J* = 34 Hz), 131.1, 130.9 (q, *J* = 3 Hz), 130.7, 127.9 (q, *J* = 4 Hz) 127.3, 123.3 (q, *J* = 273 Hz), 115.1, 68.2, 63.0, 32.8, 29.3, 26.0, 25.7; IR (KBr) *v* 3297, 2938, 2863, 1605, 1510, 1487, 1427, 1337, 1252, 1173, 1134, 1059, 1003, 908, 842, 699, 638 cm^−1^; HRMS (ESI-TOF) [M+H]^+^ *m*/*z* calcd for C_20_H_21_F_3_N_3_O_2_: 392.1586; found: 392.1590. Anal. Calcd for C_20_H_20_F_3_N_3_O_2_: C, 61.38; H, 5.15; N, 10.74. Found: C, 61.57; H, 5.12; N, 10.74.

**Preparation of triazines 2–4. A general method**. To a solution of hydrazide **8**, **9** or **10** (6.96 mmol) in glacial AcOH (100 mL), Sn powder (4.54 g, 38.3 mmol) was added and stirred at rt for 2 h, and then at 115 °C for 30 min. After cooling, EtOAc and H_2_O were added, and the mixture was filtered through a Celite pad. The solution was extracted with two portions of EtOAc, and the combined organic extracts were washed with saturated aq. NaHCO_3_ (3×) and dried (Na_2_SO_4_). Solvents were removed on a rotavap, and the residue was dissolved in a CH_2_Cl_2_/MeOH mixture (1:1) and solid NaIO_4_ (2.23 g, 10.44 mmol) or Ag_2_O (354 mg, 1.52 mmol) was added. The mixture was stirred at rt until complete consumption of the dihydro form. The solution was filtered, and the solvents were removed under reduced pressure. The crude product was purified on silica gel and recrystallized to give pure triazines **2**–**4**.

**3-(4-Acetoxymethylphenyl)-7-trifluoromethylbenzo[*e*][1,2,4]triazine (2).** Starting from hydrazide **9** using modified method. The suspension was stirred at 70 °C overnight and at 120 °C for 1 h. Solid Ag_2_O was used for oxidation. The crude product was purified by column chromatography (SiO_2_, pet. ether/AcOEt 4:1) and further by recrystallization (CH_2_Cl_2_/EtOH) to give 967 mg (40% yield) of triazine **2** as yellow crystals: mp 136–137 °C; ^1^H NMR (500 MHz, CDCl_3_) 8.84 (s, 1H), 8.76 (d, *J* = 8.3 Hz, 2H), 8.21 (d, *J* = 8.9 Hz, 1H), 8.11 (dd, *J_1_* = 8.9 Hz, *J_2_* = 1.9 Hz, 1H), 7.58 (d, *J* = 8.4 Hz, 2H), 5.23 (s, 2H), 2.16 (s, 3H). ^13^C{^1^H} NMR (151 MHz, CDCl_3_) δ 170.9, 160.6, 145.3, 142.1, 140.4, 134.8, 132.0 (q, *J* = 34 Hz), 131.1 (q, *J* = 3 Hz), 131.0, 129.5, 128.7, 128.0 (q, *J* = 4 Hz), 123.2 (q, *J* = 273 Hz), 65.8, 21.1; IR (KBr) *v* 3070, 1744, 1630, 1508, 1425, 1328, 1257, 1173, 1131, 1056, 1012, 901, 842, 641 cm^−1^; HRMS (AP-TOF) [M+H]^+^ *m*/*z* calcd for C_17_H_13_F_3_N_3_O_2_: 348.0960; found: 348.0961. Anal. Calcd for C_17_H_12_F_3_N_3_O_2_: C, 58,79; H, 3.48; N, 12.10. Found: C, 58.83; H, 3.45; N, 12.25.

**3-[3,5-Bis(hydroxymethyl)phenyl]-7-trifluoromethylbenzo[*e*][1,2,4]triazine (3).** Starting from hydrazide **10** using modified method. The suspension was stirred at rt overnight without further heating. Solid Ag_2_O was used for oxidation. The resulting crude product was purified by chromatography (SiO_2_, pet. ether/AcOEt 1:1) and further by recrystallization (AcOEt) to give 1.28 g (40–55% yield) of triazine **3** as yellow crystals: mp 174–175 °C; ^1^H NMR (500 MHz, DMSO-*d*_6_) δ 9.02 (s, 1H), 8.52 (s, 2H), 8.37 (s, 2H), 7.54 (s, 1H), 5.43 (t, *J* = 5.7 Hz, 2H), 4.66 (d, *J* = 5.6 Hz, 4H); ^13^C{^1^H} NMR (126 MHz, DMSO-*d*_6_) δ 160.1, 145.0, 143.6, 141.7, 134.3, 131.4, 131.2, 130.3 (q, *J* = 33 Hz), 128.3, 127.8, 125.1, 123.3 (q, *J* = 273 Hz), 63.2; IR (KBr) *v* 3270, 3180, 2945, 2884, 1627, 1509, 1421, 1330, 1236, 1214, 1167, 1121, 1055, 1021, 846, 664 cm^−1^; HRMS (ESI-TOF) [M+H]^+^ *m*/*z* calcd for C_16_H_13_F_3_N_3_O_2_: 336,0960; found: 336.0964. Anal. Calcd for C_16_H_12_F_3_N_3_O_2_: C, 57.32; H, 3.61; N, 12.53. Found: C, 57.39; H, 3.58; N, 12.62.

**Preparation of 3-[4-(6-benzyloxyhexyloxy)phenyl]-7-trifluoromethylbenzo[*e*][1,2,4] triazine (4)**. Starting from **8** using general method. The crude product was purified on column chromatography (SiO_2_, CH_2_Cl_2_ 100%) to give 2.50 g (75% yield) of pure triazine **4** as an orange solid: mp 109–110 °C; ^1^H NMR (500 MHz, CDCl_3_) δ 8.80 (s, 1H), 8.71 (d, *J* = 8.9 Hz, 2H), 8.14 (d, *J* = 8.9 Hz, 1H), 8.06 (dd, *J_1_* = 8.9 Hz, *J_2_* = 1.7 Hz, 1H), 7.35 (d, *J* = 4.4 Hz, 4H), 7.30–7.27 (m, 1H), 7.07 (d, *J* = 8.9 Hz, 2H), 4.52 (s, 2H), 4.08 (t, *J* = 6.5 Hz, 2H), 3.51 (t, *J* = 6.5 Hz, 2H), 1.86 (quint, *J* = 6.9 Hz, 2H), 1.68 (quint, *J* = 7.0 Hz, 2H), 1.53–1.49 (m, 4H); ^13^C{^1^H} NMR (126 MHz, CDCl_3_) δ 162.8, 160.9, 144.8, 142.3, 138.7, 131.2 (q, *J* = 34 Hz), 131.1, 130.8 (q, *J* = 2 Hz), 130.7, 128.5, 127.9 (q, *J* = 3 Hz), 127.8, 127.6, 127.2, 123.3 (q, *J* = 273), 115.1, 73.0, 70.4, 68.3, 29.8, 29.3, 26.1, 26.0; IR (KBr) *v* 2939, 2849, 1603, 1509, 1426, 1322, 1258, 1178, 1128, 1089, 1055, 977, 842 cm^−1^; HRMS (ESI-TOF) [M+H]^+^ *m*/*z* calcd for C_27_H_27_F_3_N_3_O_2_: 482.2055; found: 482.2050. Anal. Calcd for C_27_H_26_F_3_N_3_O_2_: C, 67.35; H, 5.44; N, 8.73. Found: C, 67.32; H, 5.56; N, 8.50.

**Preparation of hydrazides 5–7. A general method**. The solution of methyl benzoate derivative (4.00 g, 24.0 mmol) and hydrazine monohydrate (8.0 mL) in EtOH (100 mL) was refluxed for 48 h. The reaction mixture was cooled and concentrated in vacuo. Crude hydrazides **6** and **7** were recrystallized from hot EtOH to give pure products as white crystals. The crude hydrazide **5** was dissolved in EtOAc, washed with H_2_O and dried (Na_2_SO_4_). Solvents were removed on rotavap, and the product was purified by column chromatography (SiO_2_, CH_2_Cl_2_/EtOAc 4:1 gradient to 1:3).

**4-(6-Benzyloxyhexyloxy)benzhydrazide (5)**. Yield: 5.43 g (70% yield) of pure product **5** as a colorless solid: mp 88–89 °C; ^1^H NMR (500 MHz, DMSO-*d*_6_) δ 9.61 (s, 1H), 7.78 (d, *J* = 8.8 Hz, 2H), 7.35–7.30 (m, 4H), 7.27 (t, *J* = 6.9 Hz, 1H), 6.95 (d, *J* = 8.8 Hz, 2H), 4.44 (s, 4H), 3.99 (t, *J* = 6.5 Hz, 2H), 3.42 (t, *J* = 6.5 Hz, 2H), 1.70 (quint, *J* = 6.8 Hz, 2H), 1.55 (quint, *J* = 6.8 Hz, 2H), 1.44–1.34 (m, 4H); ^13^C{^1^H} NMR (126 MHz, DMSO-*d*_6_) δ 165.6, 160.9, 138.7, 128.7, 128.2, 127.4, 127.3, 125.3, 114.0, 71.8, 69.5, 67.6, 29.2, 28.6, 25.5, 25.3; IR (KBr) v 3321, 2940, 2852, 2796, 1618, 1605, 1536, 1500, 1455, 1341, 1258, 1175, 1127, 1105, 1026, 942, 846, 740 cm^−1^; HRMS (ESI-TOF) [M+H]^+^ *m*/*z* calcd for C_20_H_27_N_2_O_3_: 343.2022; found: 343.2027. Anal. Calcd for C_20_H_26_N_2_O_3_: C, 70.15; H, 7.65; N, 8.18. Found: C, 70.10; H, 7.60; N, 8.36.

**4-Hydroxymethylbenzhydrazide (6).** Yield: 3.80 g (95% yield) of **6** as colorless crystals: ^1^H NMR (500 MHz, DMSO-*d*_6_) δ 9.73 (s, 1H), 7.78 (d, *J* = 8.3 Hz, 2H), 7.37 (d, *J* = 8.4 Hz, 2H), 5.30 (t, *J* = 5.7 Hz, 1H), 4.54 (d, *J* = 5.2 Hz, 2H), 4.48 (s, 2H); ^13^C{^1^H} NMR (126 MHz, DMSO-*d*_6_) δ 166.0, 145.8, 131.7, 126.9, 126.1, 62.5; HRMS (ESI-TOF) [M+H]^+^ *m*/*z* calcd for C_8_H_11_N_2_O_2_: 167.0821; found: 167.0821. Anal. Cald for C_8_H_10_N_2_O_2_: C, 57.82; H, 6.07; N, 16.86. Found: C, 57.84; H, 6.04; N, 16.89. 

**3,5-Bis(hydroxymethyl)benzhydrazide (7).** Yield: 4.24 g (90% yield) of **7** as colorless crystals: mp 173–174 °C; ^1^H NMR (500 MHz, DMSO-*d*_6_) δ 9.72 (s, 1H), 7.63 (s, 2H), 7.42 (s, 1H), 5.28 (t, *J* = 5.7 Hz, 2H), 4.52 (d, *J* = 5.4 Hz, 4H), 4.48 (s, 2H); ^13^C{^1^H} NMR (126 MHz, DMSO-*d*_6_) δ 166.2, 142.5, 133.1, 127.2, 123.6, 62.8; IR (KBr) *v* 3263, 3149, 3051, 2929, 2872, 2783, 2664, 1638, 1536, 1460, 1355, 1260, 1156, 1045, 874, 704 cm^−1^; HRMS (ESI-TOF) [M+H]^+^ *m*/*z* calcd for C_9_H_13_N_2_O_3_: 197.0926; found: 197.0930. Anal. Calcd for C_9_H_12_N_2_O_3_: C, 55.09; H, 6.16; N, 14.28. Found: C, 55.08; H, 6.11; N, 14.03.

**Preparation of hydrazides 8–10. A general method.** To a solution of substituted benzhydrazide **5**–**7** (8.76 mmol) in dry DMSO (20 mL), 1-fluoro-2-nitro-5-trifluoromethylbenzene (1.83 g, 8.76 mmol) was added, and the reaction mixture was stirred at 70 °C overnight. The solution was cooled to rt and poured into a large amount of H_2_O and extracted with EtOAc. The organic layer was separated, washed with H_2_O (3x), then with brine, and dried (Na_2_SO_4_). Solvents were removed under vacuum and the crude product was recrystallized twice from EtOH to give corresponding pure hydrazide **5**–**7** as an orange solid.

***N*-(2-nitro-5-trifluoromethylbenzene)-4-(6-benzyloxyhexyloxy) benzhydrazide (8)**. From hydrazide **5**. Yield: 3.85 g (83% yield) of **8** as an orange solid: mp 115–116 °C; ^1^H NMR (500 MHz, DMSO-*d*_6_) δ 10.74 (s, 1H), 9.69 (s, 1H), 8.32 (d, *J* = 8.8 Hz, 1H), 7.92 (d, *J* = 8.8 Hz, 2H), 7.34–7.30 (m, 5H), 7.27 (t, *J* = 6.8 Hz, 1H), 7.17 (dd, *J*_1_ = 8.8 Hz, *J*_2_ = 1.6 Hz, 1H), 7.06 (d, *J* = 8.8 Hz, 2H), 4.44 (s, 2H), 4.04 (t, *J* = 6.4 Hz, 2H), 3.43 (t, *J* = 6.4 Hz, 2H), 1.74 (quint, *J* = 6.8 Hz, 2H), 1.57 (quint, *J* = 6.8 Hz, 2H), 1.46–1.36 (m, 4H); ^13^C{^1^H} NMR (126 MHz, DMSO-*d*_6_) δ 165.7, 161.9, 145.3, 138.8, 135.2 (q, *J* = 32 Hz), 133.5, 129.5, 128.2, 127.9, 127.4, 127.3, 123.9, 123.1 (q, *J* = 274 Hz), 114.4, 113.4, 111.8, 71.8, 69.6, 67.8, 29.2, 28.5, 25.5, 25.3; IR (KBr) *v* 3319, 3253, 2933, 2854, 1642, 1607, 1540, 1493, 1436, 1339, 1297, 1256, 1227, 1174, 1128, 937, 892, 844, 765, 731, 697 cm^−1^; HRMS (ESI-TOF) [M+H]^+^ *m*/*z* calcd for C_27_H_29_F_3_N_3_O_5_: 532.2059; found: 532.2062. Anal. Calcd for C_27_H_28_F_3_N_3_O_5_: C, 61.01; H, 5.31; N, 7.91. Found: C, 61.01; H, 5.36; N, 8.14.

***N*’-(4-(hydroxymethyl)phenyl)-2-nitro-5-(trifluoromethyl) benzhydrazide (9).** From hydrazide **6**. Yield: 3.05 g (98% yield) of **9** as yellow crystals: mp 189–190 °C; ^1^H NMR (500 MHz, DMSO-*d*_6_) δ 10.86 (s, 1H), 9.72 (s, 1H), 8.32 (d, *J* = 8.8 Hz, 1H), 7.92 (d, *J* = 8.3 Hz, 2H), 7.49 (d, *J* = 8.3 Hz, 2H), 7.35 (d, *J* = 1.2 Hz, 1H), 7.18 (dd, *J_1_* = 8.8 Hz, *J_2_* = 1.8 Hz, 1H), 5.37 (t, *J* = 5.7 Hz, 1H), 4.59 (d, *J* = 5.7 Hz, 2H); ^13^C{^1^H} NMR (126 MHz, DMSO-*d*_6_) δ 166.2, 147.2, 145.2, 135.2 (q, *J* = 32 Hz), 133.6, 130.3, 127.9, 127.4, 126.4, 123.1 (q, *J* = 273 Hz), 113.4, 111.8, 62.4; IR (KBr) *v* 3325, 3256, 1648, 1594, 1536, 1492, 1339, 1263, 1228, 1190, 1127, 1056, 935, 831, 763 cm^−1^; HRMS (ESI-TOF) [M+H]^+^ *m*/*z* calcd for C_15_H_13_F_3_N_3_O_4_: 356.0858; found: 356.0849. Anal. Calcd for C_15_H_12_F_3_N_3_O_4_: C, 50.71; H, 3.40; N, 11.83. Found: C, 50.78; H, 3.39; N, 12.00.

***N*’-(3,5-bis(hydroxymethyl)phenyl)-2-nitro-5-(trifluoromethyl)benzohydrazide (10).** From hydrazide **7**. Yield: 3.04 g (90% yield) of **10** as yellow crystals: mp 195–196 °C; ^1^H NMR (500 MHz, DMSO-*d*_6_) δ 10.89 (s, 1H), 9.73 (s, 1H), 8.33 (d, *J* = 8.6 Hz, 1H), 7.79 (s, 2H), 7.53 (s, 1H), 7.33 (d, *J* = 1.1 Hz, 1H), 7.17 (dd, *J_1_* = 8.9 Hz, *J_2_* = 1.7 Hz, 1H), 5.37 (t, *J* = 5.7 Hz, 2H), 4.59 (d, *J* = 5.6 Hz, 4H); ^13^C{^1^H} NMR (126 MHz, DMSO-*d*_6_) δ 166.5, 145.2, 143.0, 135.2 (q, *J* = 32 Hz), 133.6, 131.8, 128.2, 128.0, 123.9, 123.2 (q, *J* = 273 Hz), 113.4, 111.7, 62.6; IR (KBr) *v* 3260, 2952, 1648, 1590, 1489, 1339, 1263, 1182, 1130, 1057, 987, 882; HRMS (ESI-TOF) [M+H]^+^ *m*/*z* calcd for C_16_H_15_F_3_N_3_O_5_: 386,0964; found: 386.0962. Anal. Calcd for C_16_H_14_F_3_N_3_O_5_: C, 49.88; H, 3.66; N, 10.91. Found: C, 49.90; H, 3.47; N, 11.04.

## Data Availability

Data obtained in this project are contained within this article and available upon request from the Authors.

## Supplementary Materials

The following are available online: NMR (^1^H and ^13^C{^1^H}; Figures S1–S18), UV (Figures S19–S22) and EPR spectra (Figures S23–S30). The supporting information can be downloaded.

## Sample Availability

Samples of the compounds are not available from the authors.
