# Supplementary material for: Tethered Blatter Radical for Molecular Grafting: Synthesis of 6-Hydroxyhexyloxy, Hydroxymethyl, and Bis(hydroxymethyl) Derivatives and Their Functionalization
Source: Molecules. 2022 Feb 9;27(4):1176. doi: 10.3390/molecules27041176 (PMC8876519; doi:10.3390/molecules27041176)
Supplement: Supplementary file 1 [file molecules-27-01176-s001.zip › molecules-1576031-supplementary.pdf]

Supporting Information

for

Tethered Blatter radical for molecular grafting: Synthesis of 6-hydroxyhexyloxy, hydroxymethyl, and bis(hydroxymethyl) derivatives and their functionalization

Szymon Kapuściński [1,2], Bindushree Anand [2], Paulina Bartos [1], Jose M. Garcia Fernandez [3*], and Piotr Kaszyński [1,2,4 *]

[1] Faculty of Chemistry, University of Łódź, Tamka 12, 91-403 Łódź, Poland

[2] Centre for Molecular and Macromolecular Studies, Polish Academy of Sciences, Sienkiewicza 112, 90-363 Łódź, Poland

[3] Institute fir Chemical Research, CSIC – University of Sevilla, Americo Vespucio 49, Isla de la Cartuja 41092 Sevilla, Spain

[4] Department of Chemistry, Middle Tennessee State University, Murfreesboro, TN 37-132, USA.

Table of Contents **Page**

1.	NMR spectra	S2
2.	UV-vis absorption spectroscopy	S11
3.	EPR spectroscopy	S14

1. NMR spectra

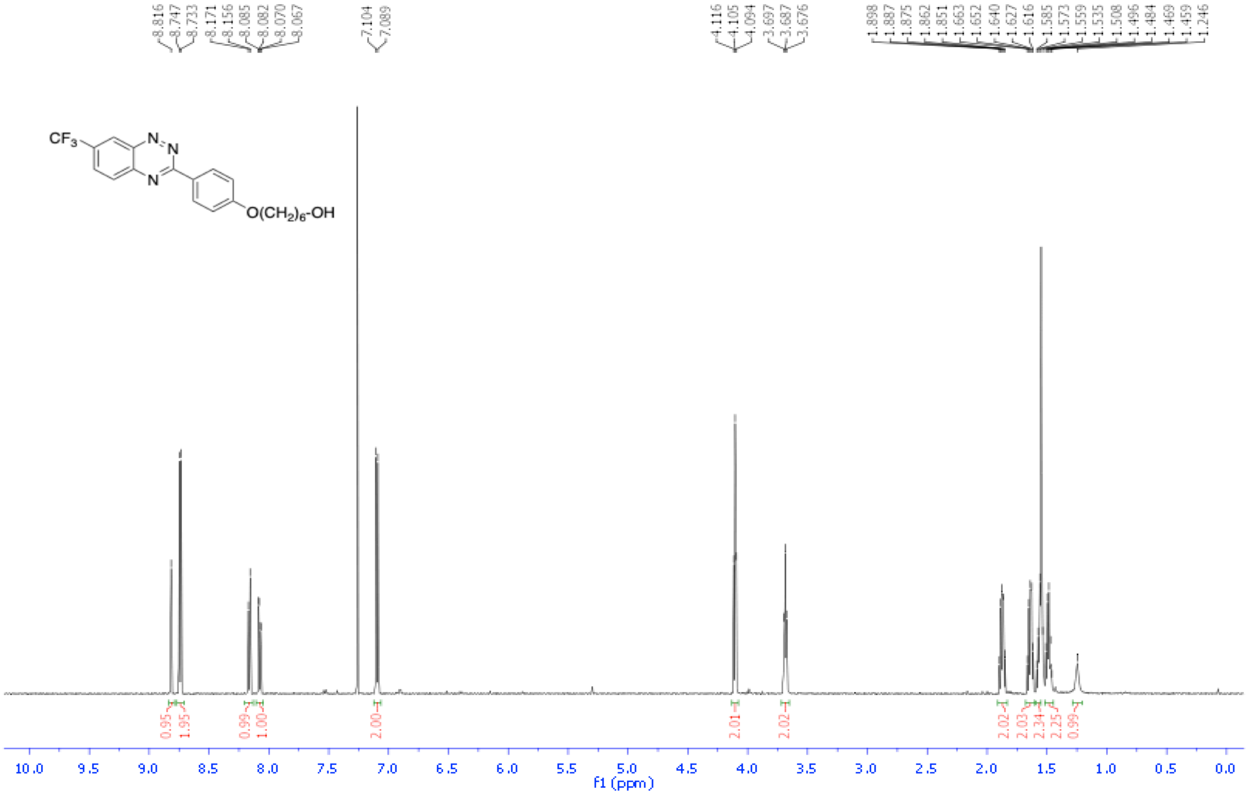

Figure S1. ^{1}H NMR spectrum for **1** recorded in $CDCl_3$.

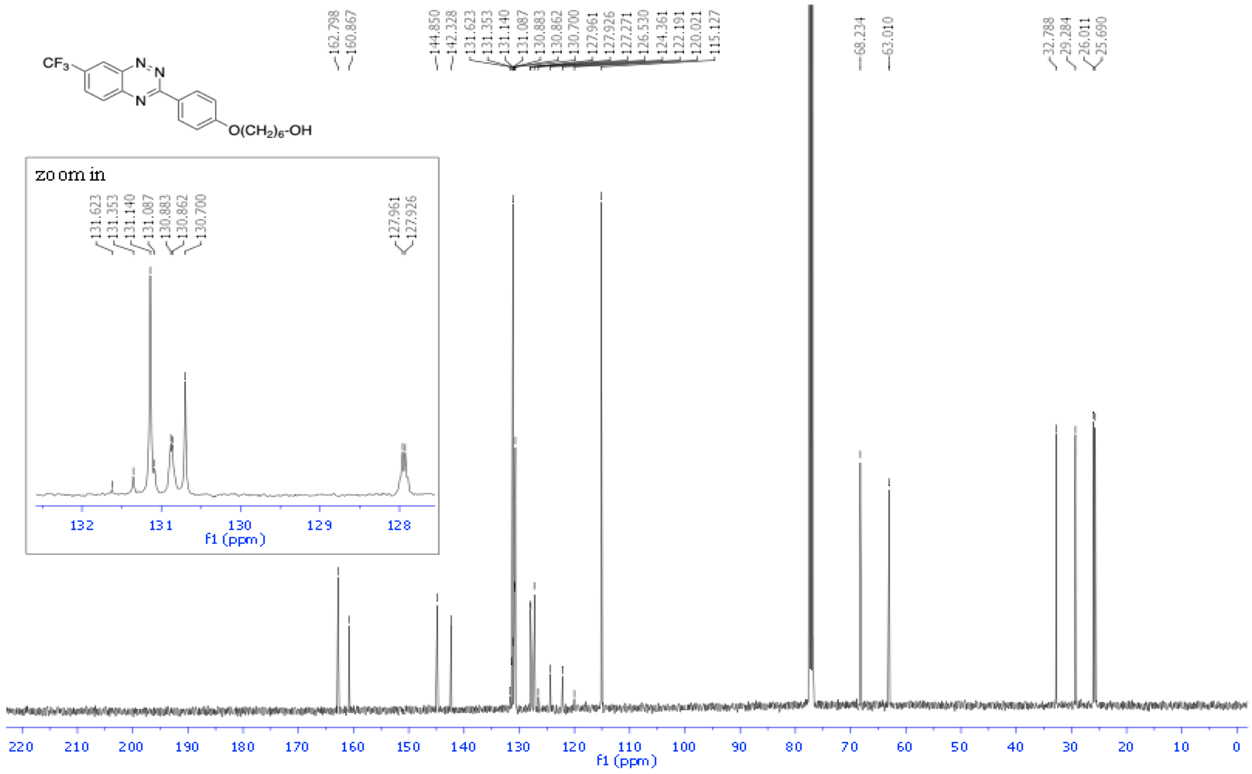

Figure S2. $^{13}C\{^{1}H\}$ NMR spectrum for **1** recorded in $CDCl_3$.

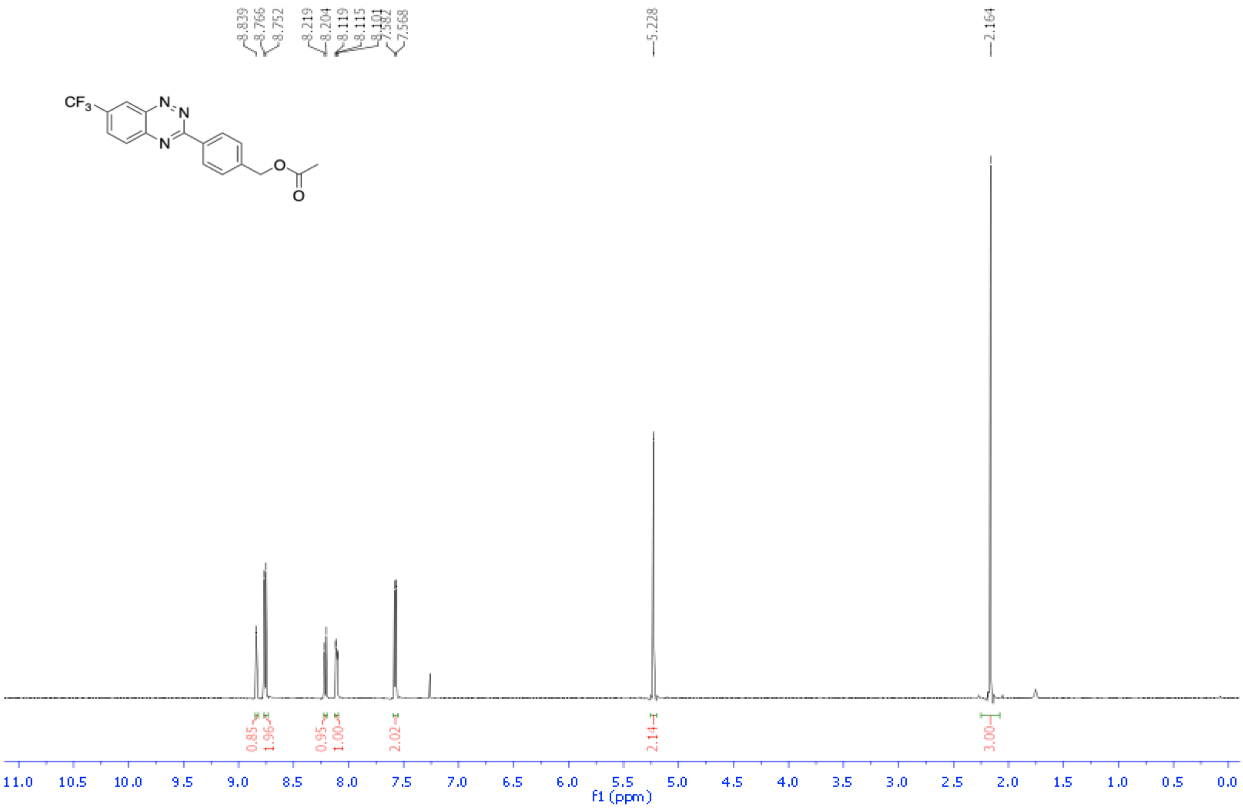

Figure S3. ^{1}H NMR spectrum for **2** recorded in $CDCl_3$.

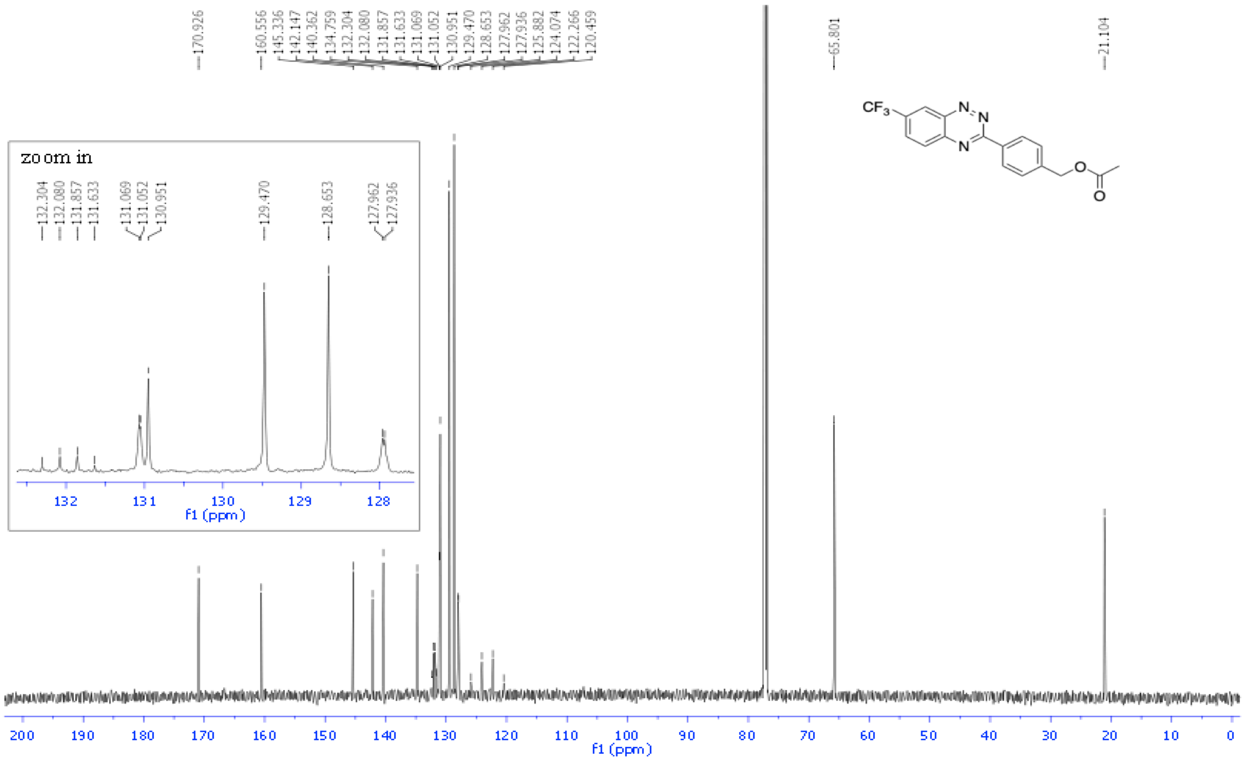

Figure S4. ${}^{13}C\{{}^{1}H\}$ NMR spectrum for **2** recorded in $CDCl_3$.

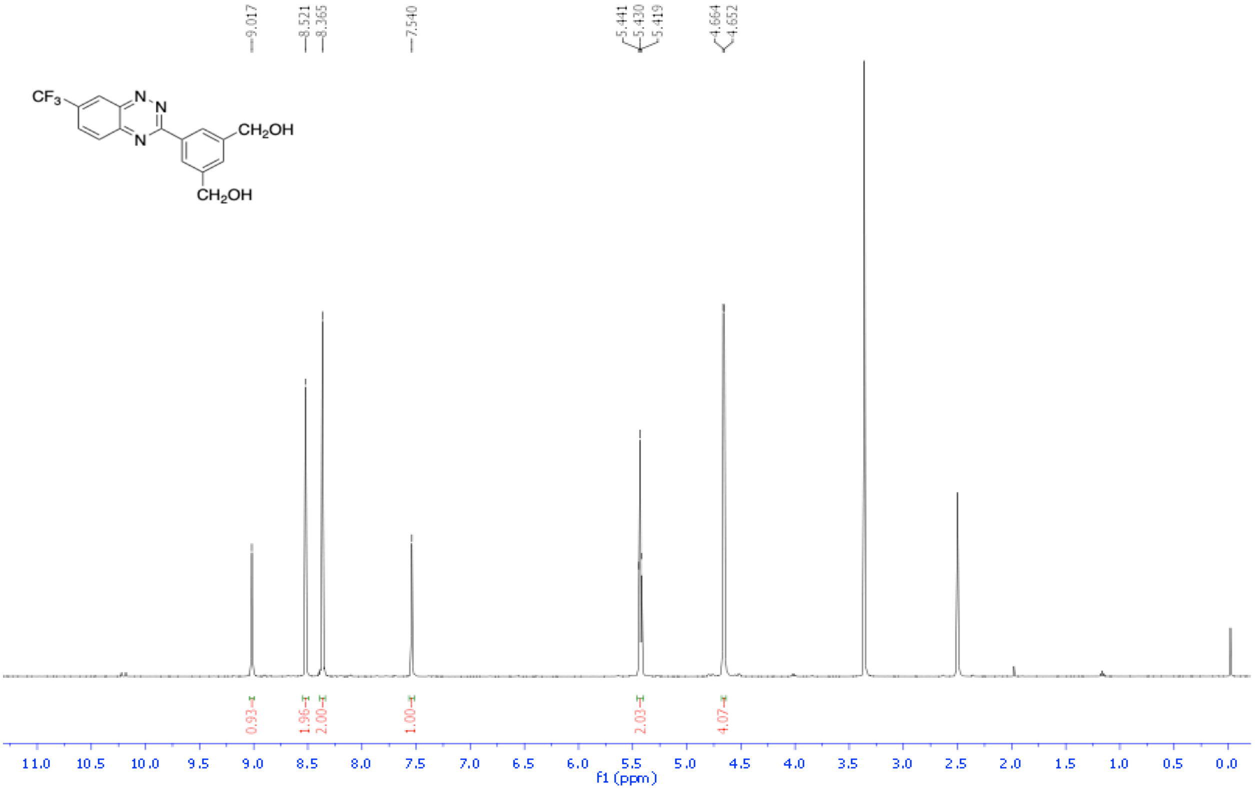

Figure S5. ^{1}H NMR spectrum for **3** recorded in DMSO-d_6.

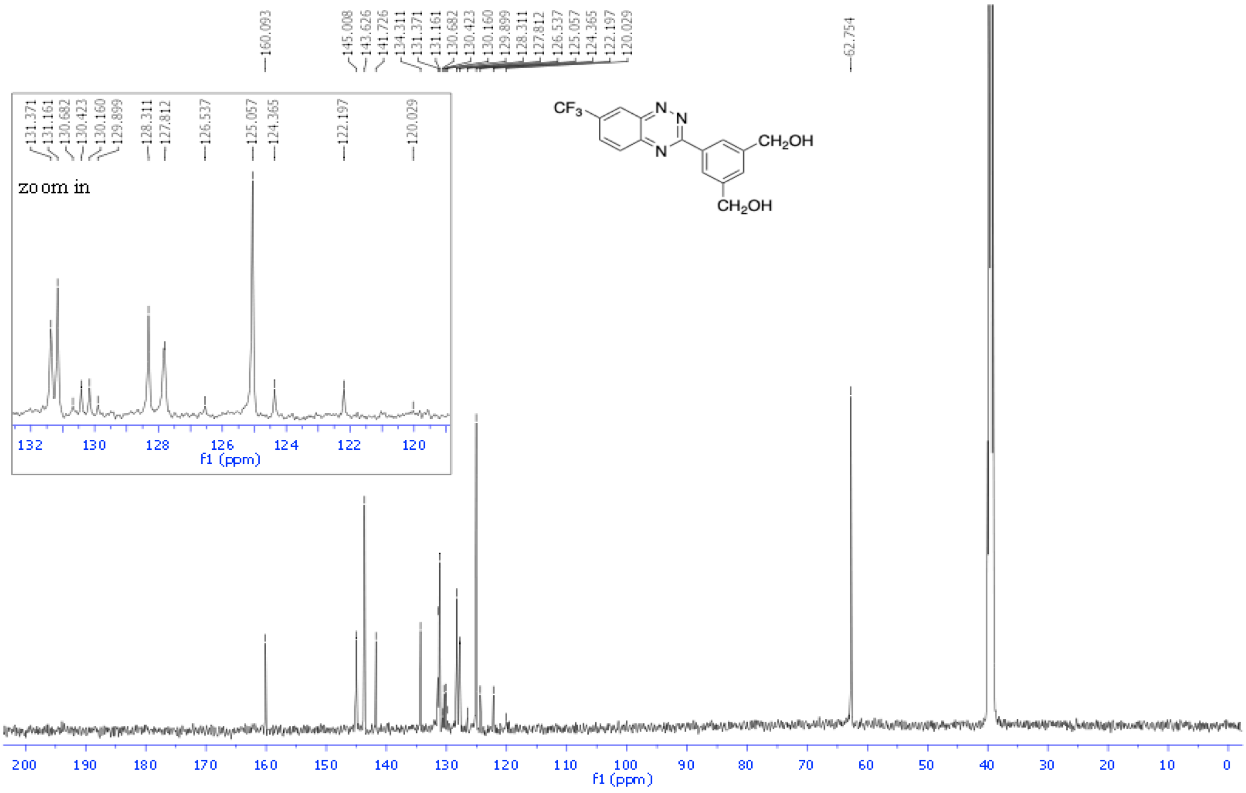

Figure S6. ^{13}C{^{1}H} NMR spectrum for **3** recorded in DMSO-d_6.

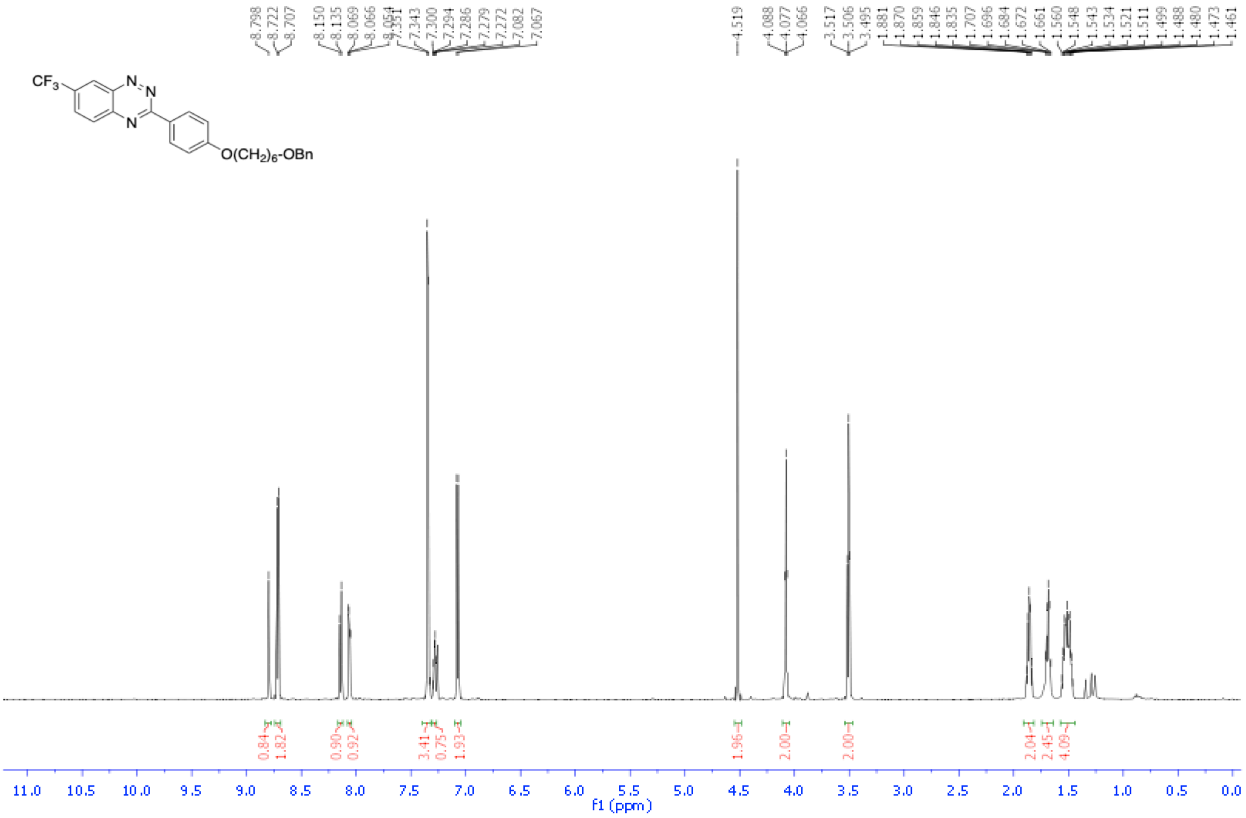

Figure S7. ^{1}H NMR spectrum for **4** recorded in $CDCl_3$.

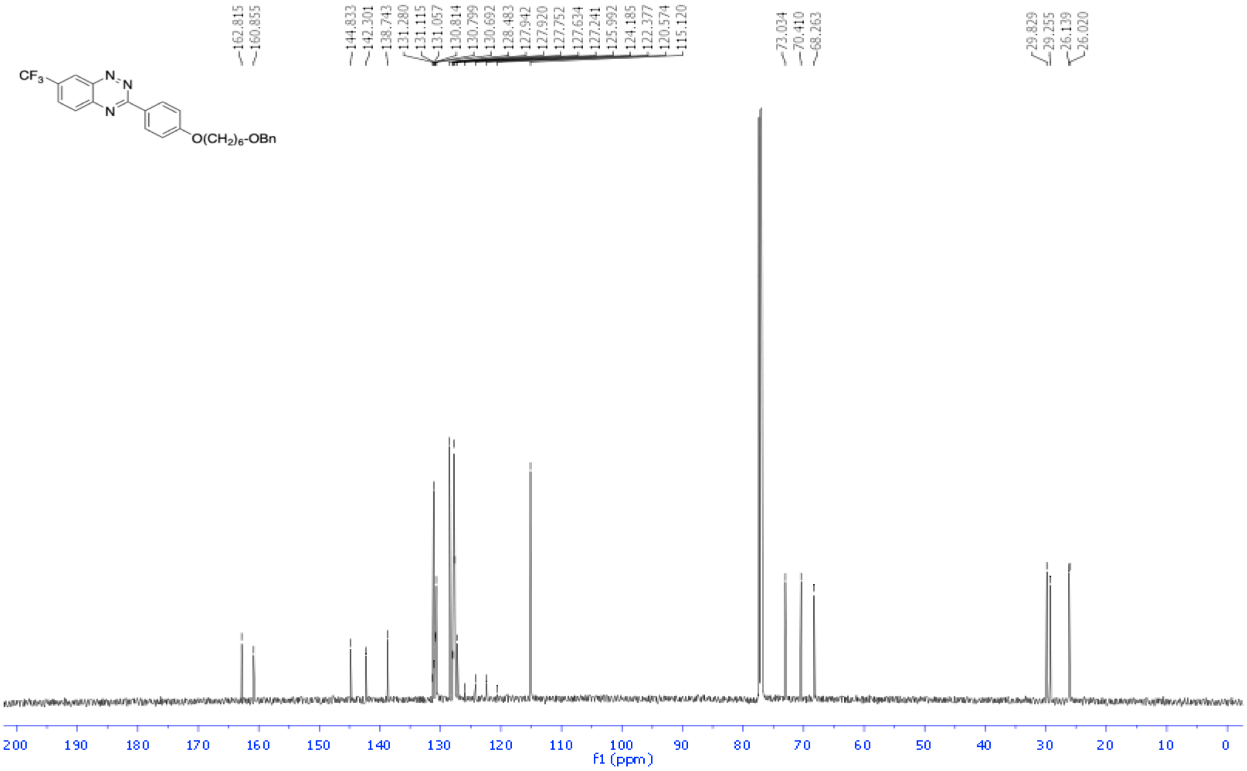

Figure S8. $^{13}C\{^{1}H\}$ NMR spectrum for **4** recorded in $CDCl_3$.

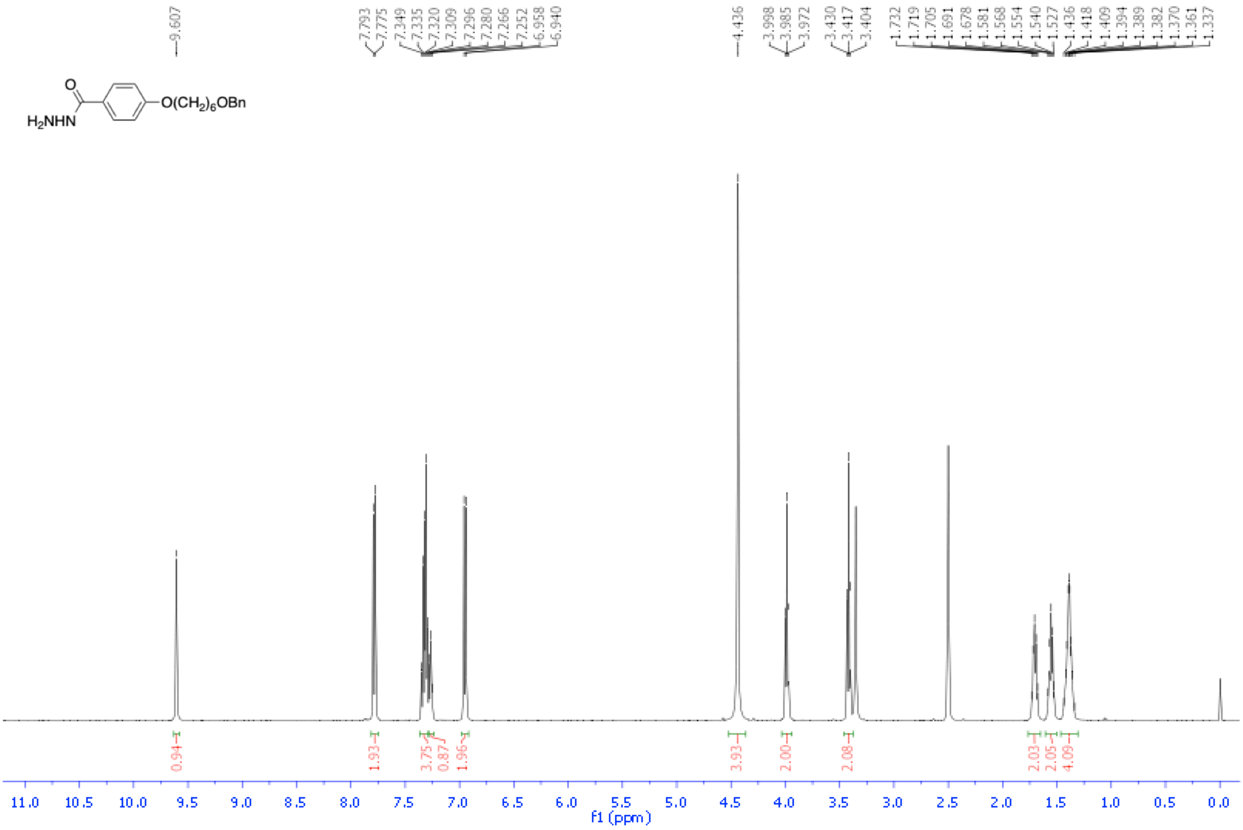

Figure S9. ^{1}H NMR spectrum for **5** recorded in DMSO-d_6.

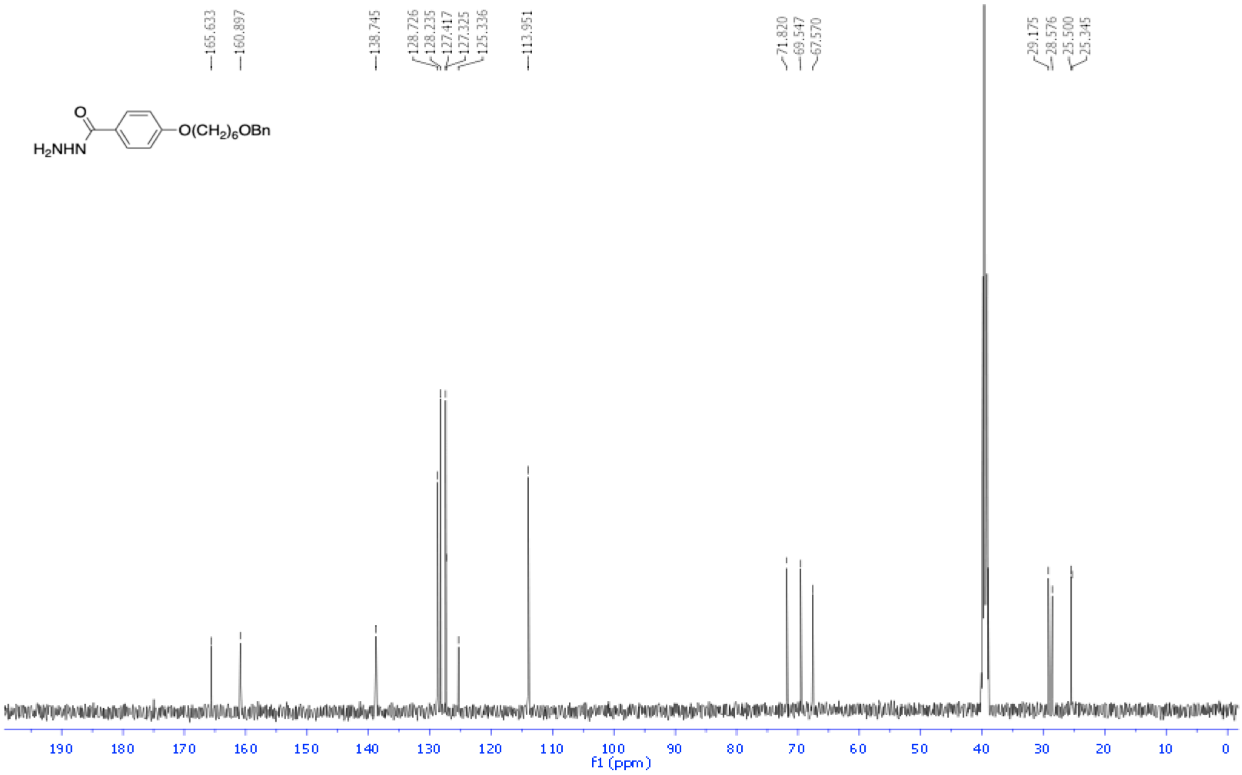

Figure S10. $^{13}C\{^1H\}$ NMR spectrum for **5** recorded in DMSO-d_6.

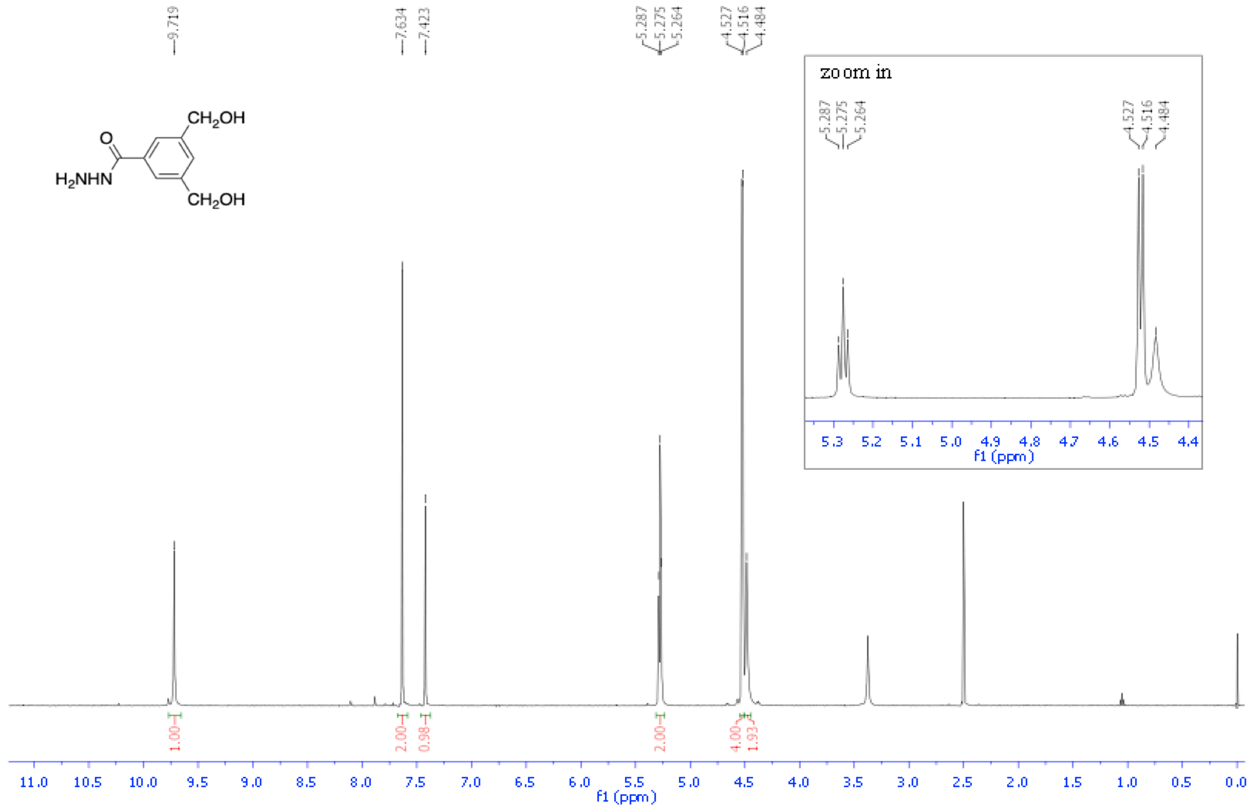

Figure S11. ^{1}H NMR spectrum for **7** recorded in DMSO-d_6.

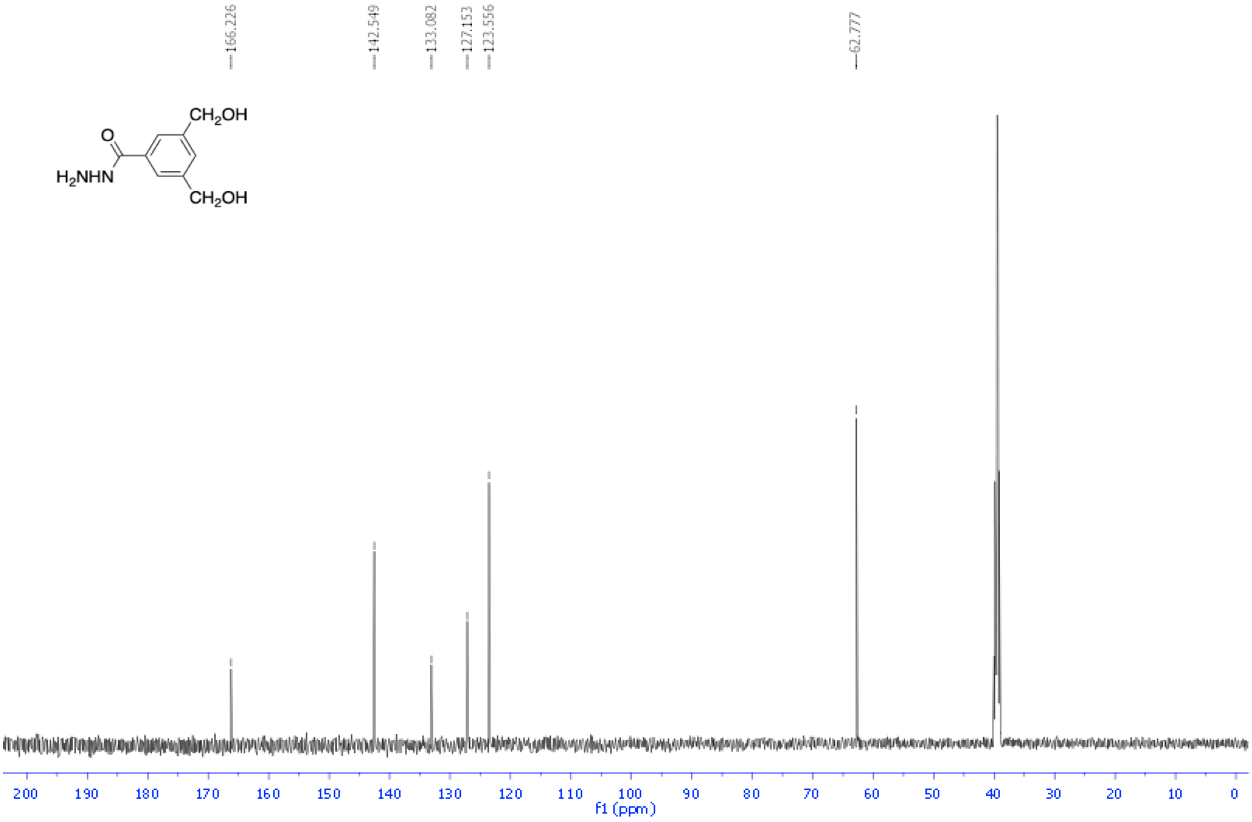

Figure S12. ^{13}C{^{1}H} NMR spectrum for **7** recorded in DMSO-d_6.

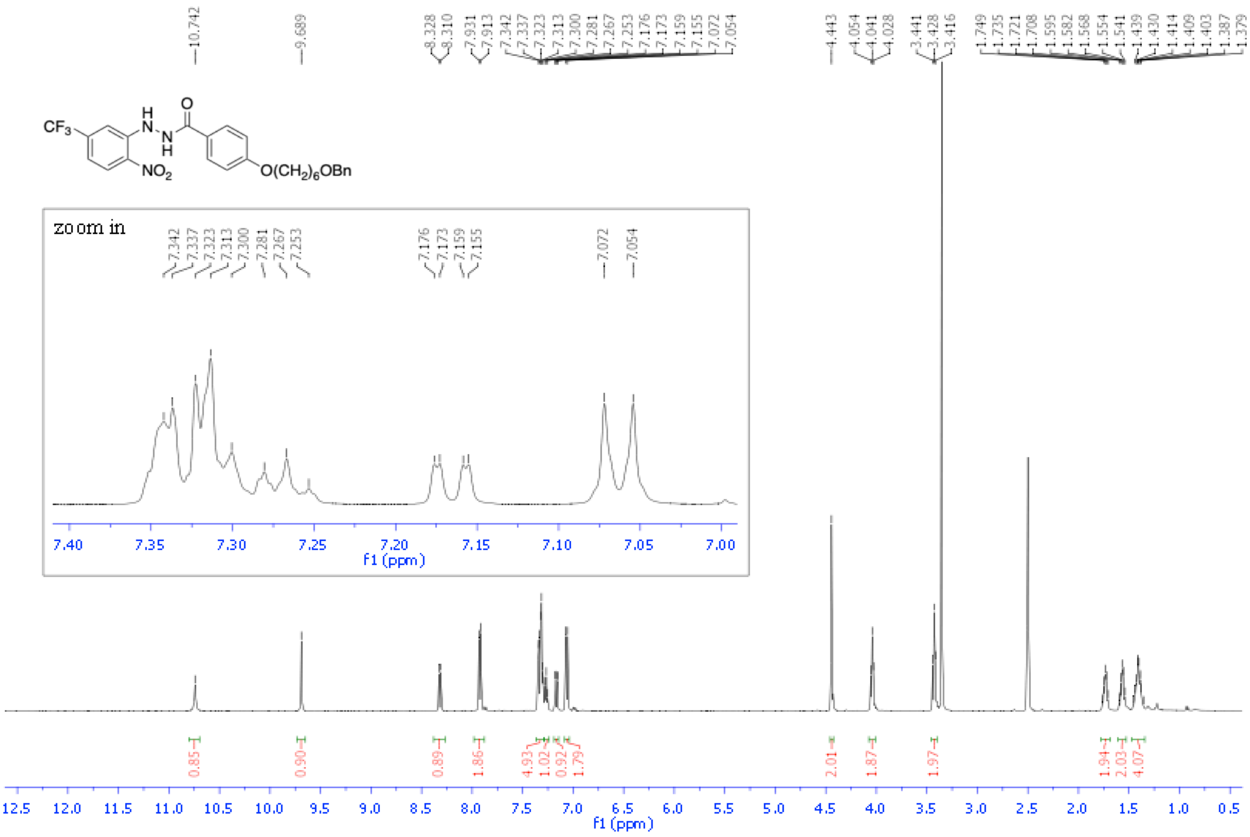

Figure S13. $^{13}C\{^{1}H\}$ NMR spectrum for **8** recorded in DMSO-d_6.

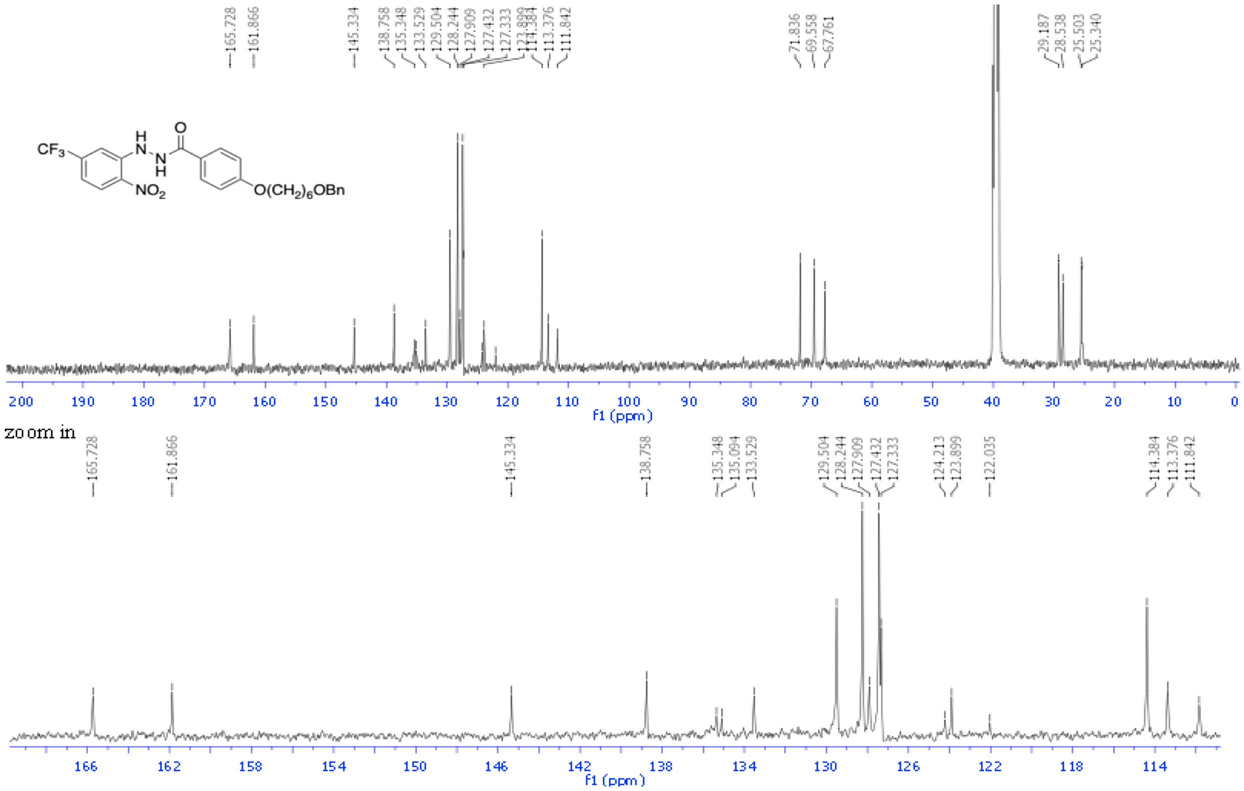

Figure S14. $^{13}C\{^{1}H\}$ NMR spectrum for **8** recorded in DMSO-d_6.

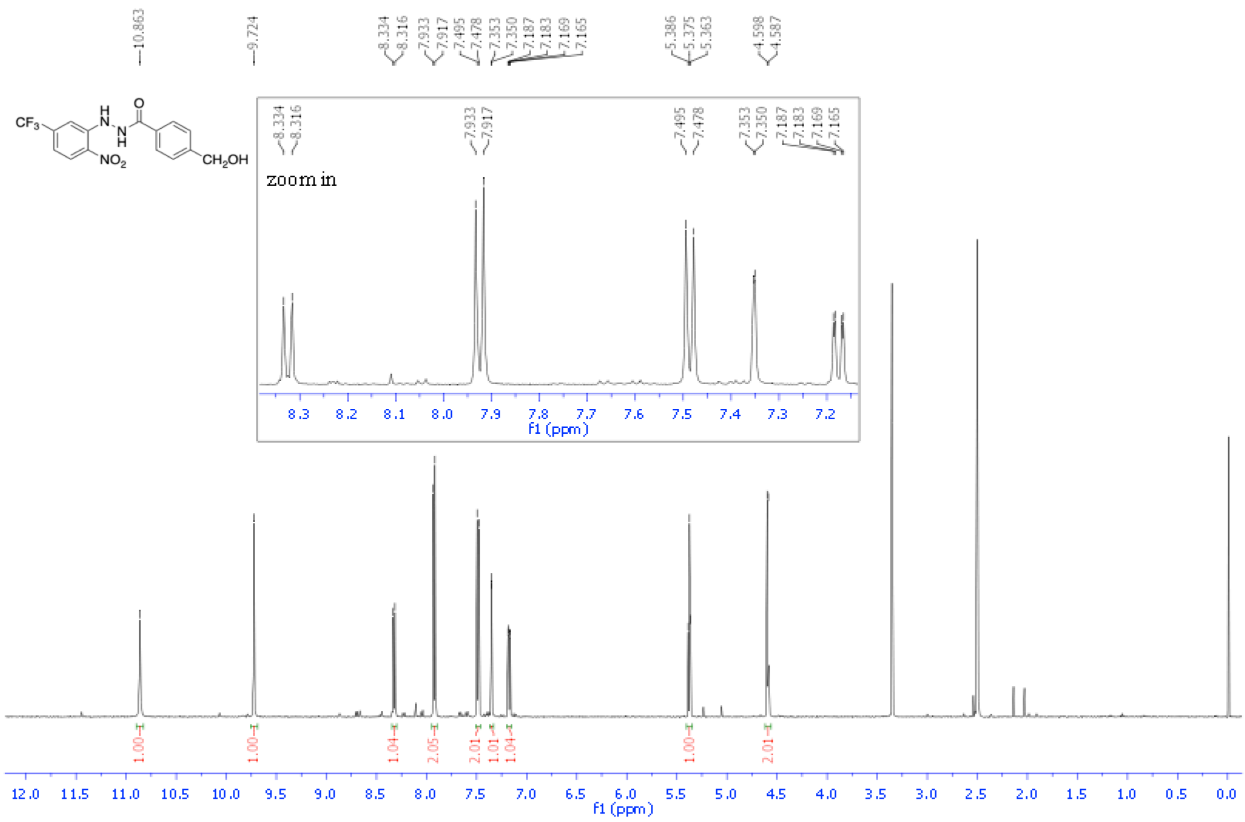

Figure S15. ^{1}H NMR spectrum for **9** recorded in DMSO-d_6.

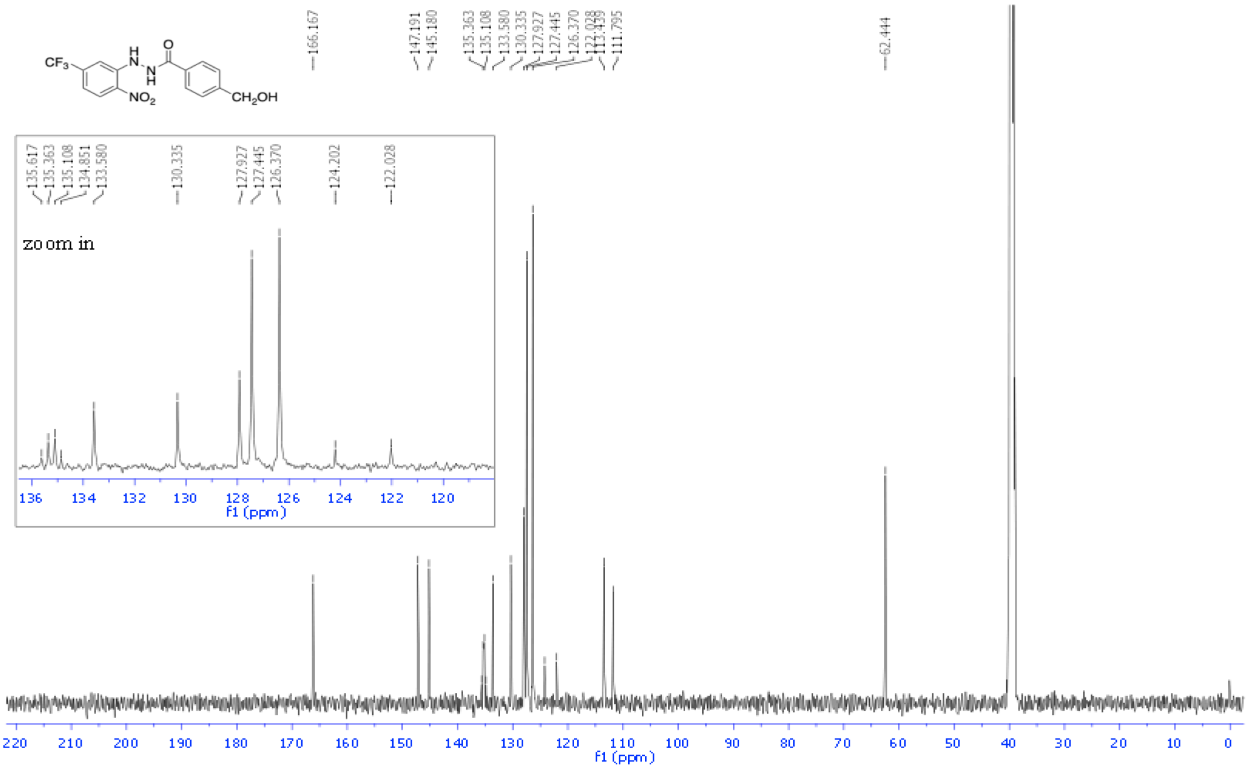

Figure S16. $^{13}C\{^{1}H\}$ NMR spectrum for **9** recorded in DMSO-d_6.

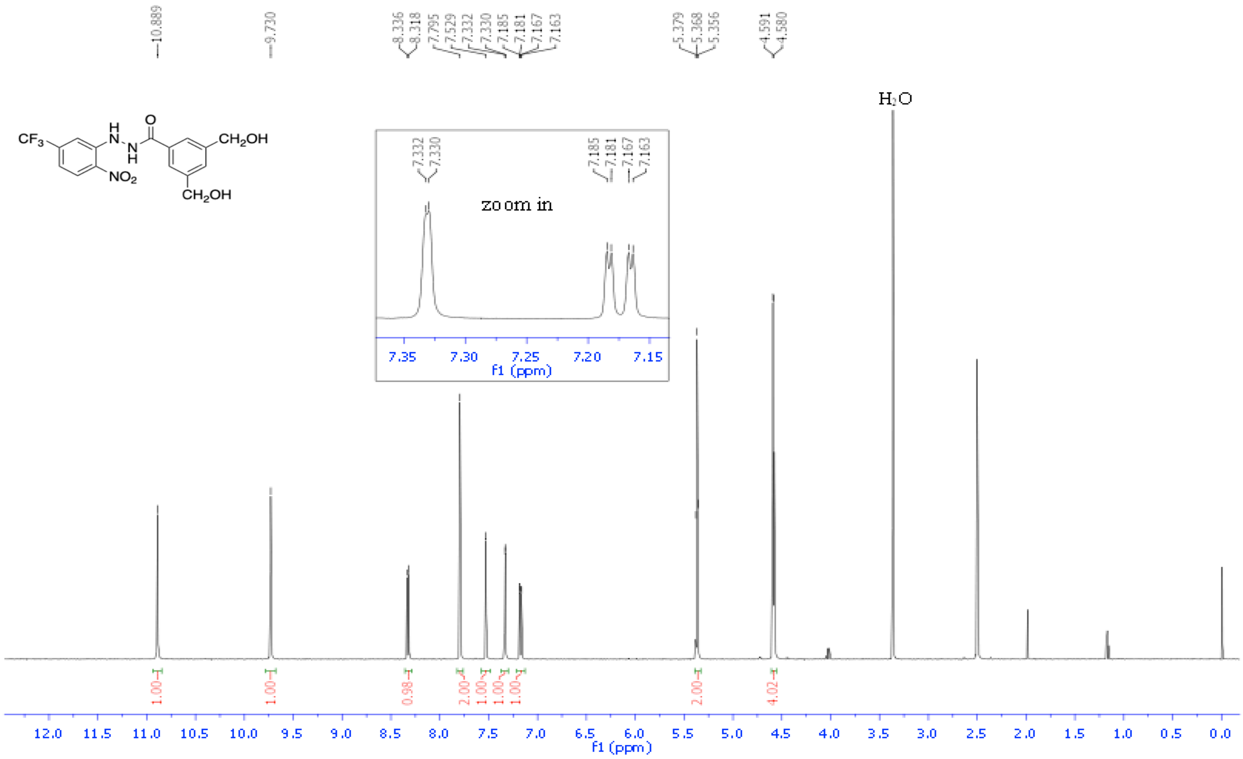

Figure S17. ^{1}H NMR spectrum for **10** recorded in DMSO-d_6.

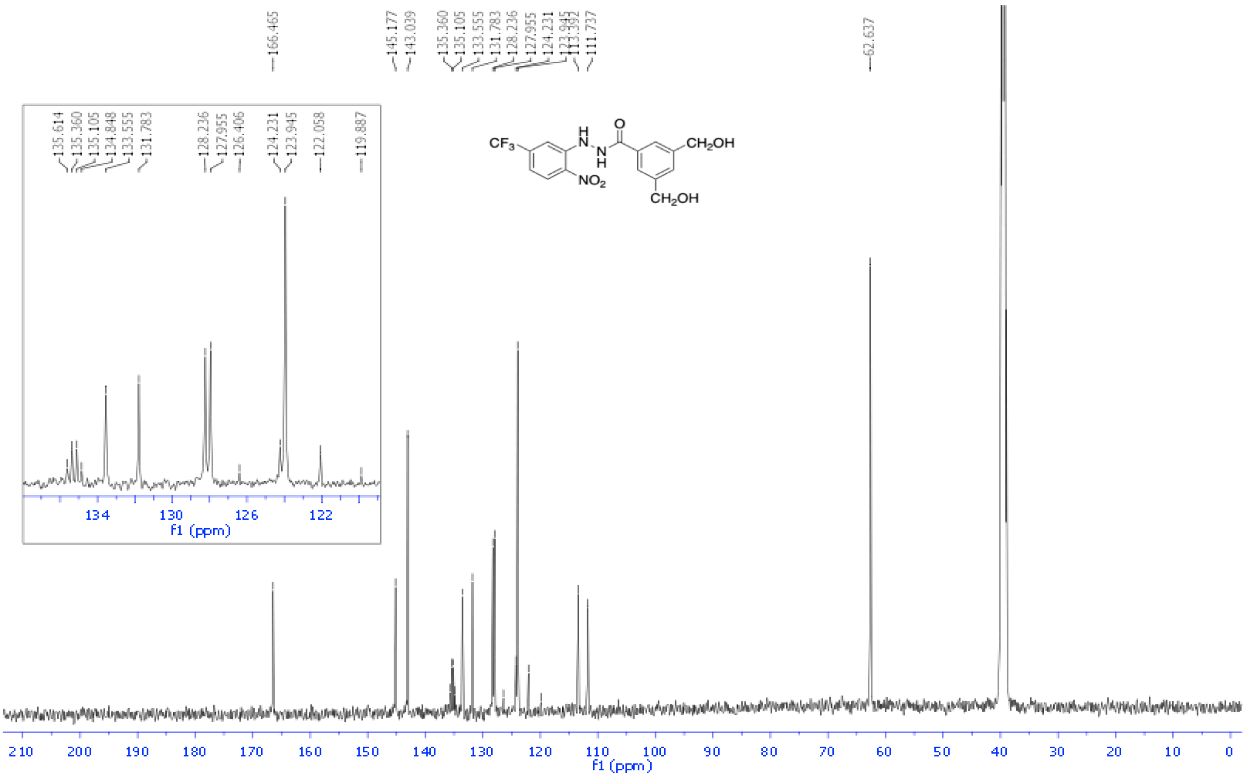

Figure S18. $^{13}C\{^1H\}$ NMR spectrum for **10** recorded in DMSO-d_6.

2. UV-vis absorption spectroscopy

Electronic absorption spectra for radicals **I** and **II** were recorded on Jasco V-770 UV-Vis-NIR spectrometer in spectroscopic grade CH_2Cl_2 at concentrations in a range 0.4–10×10^{-5} and fitted to the Beer–Lambert law. Molar extinction coefficient ε was determined based on the most intense absorption band from the equation: $\varepsilon = \varepsilon_{mol} \times concentration$. Molar extinction log ($\varepsilon$) plot was prepared for the highest concentration based on the equation: $\log \varepsilon = \log(\frac{Abs \times \varepsilon_{mol}}{Abs_{max}})$. Results are shown in Figures S19–S22.

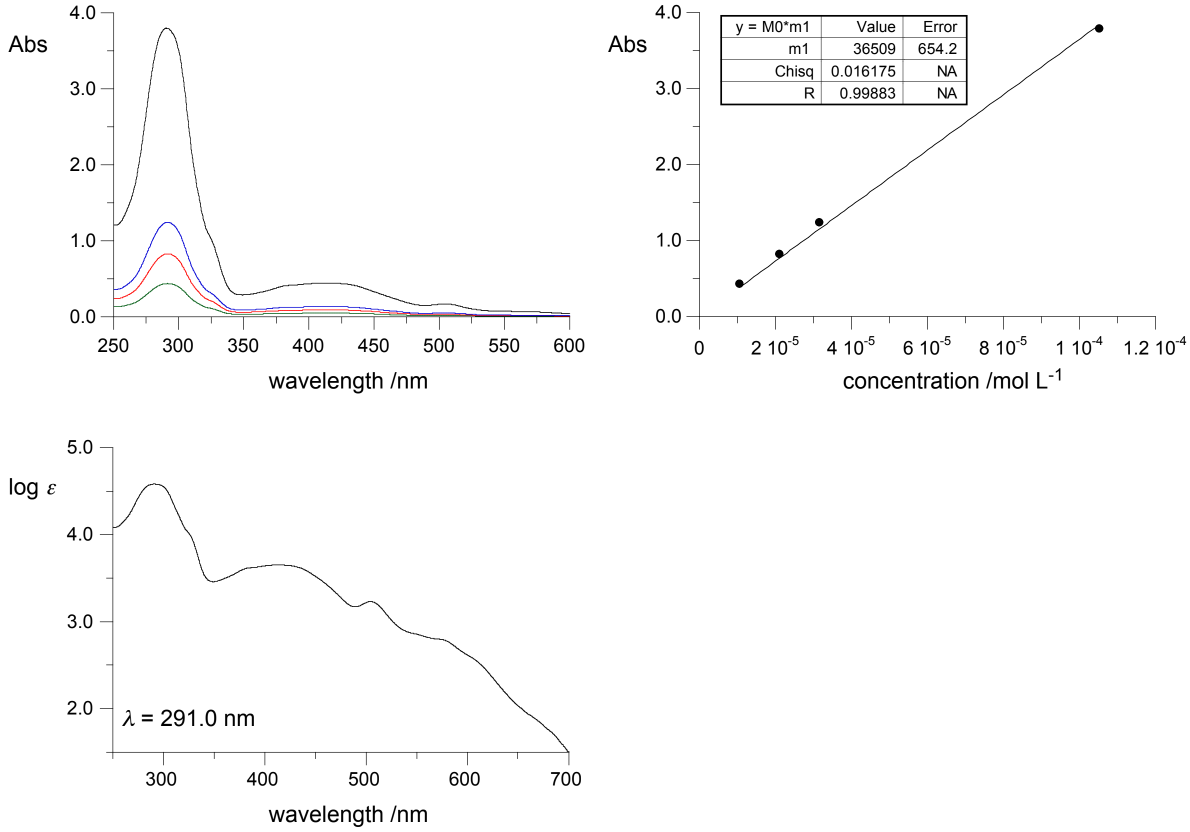

Figure S19. UV-vis absorption spectra of for **Ia** recorded in CH_2Cl_2 at four different concentrations (top left), determination of molar extinction coefficient ε at λ = 291.0 nm (top right, best fit function: ε = 36509×conc, r^2 = 0.9988) and molar extinction log (ε) plot (bottom).

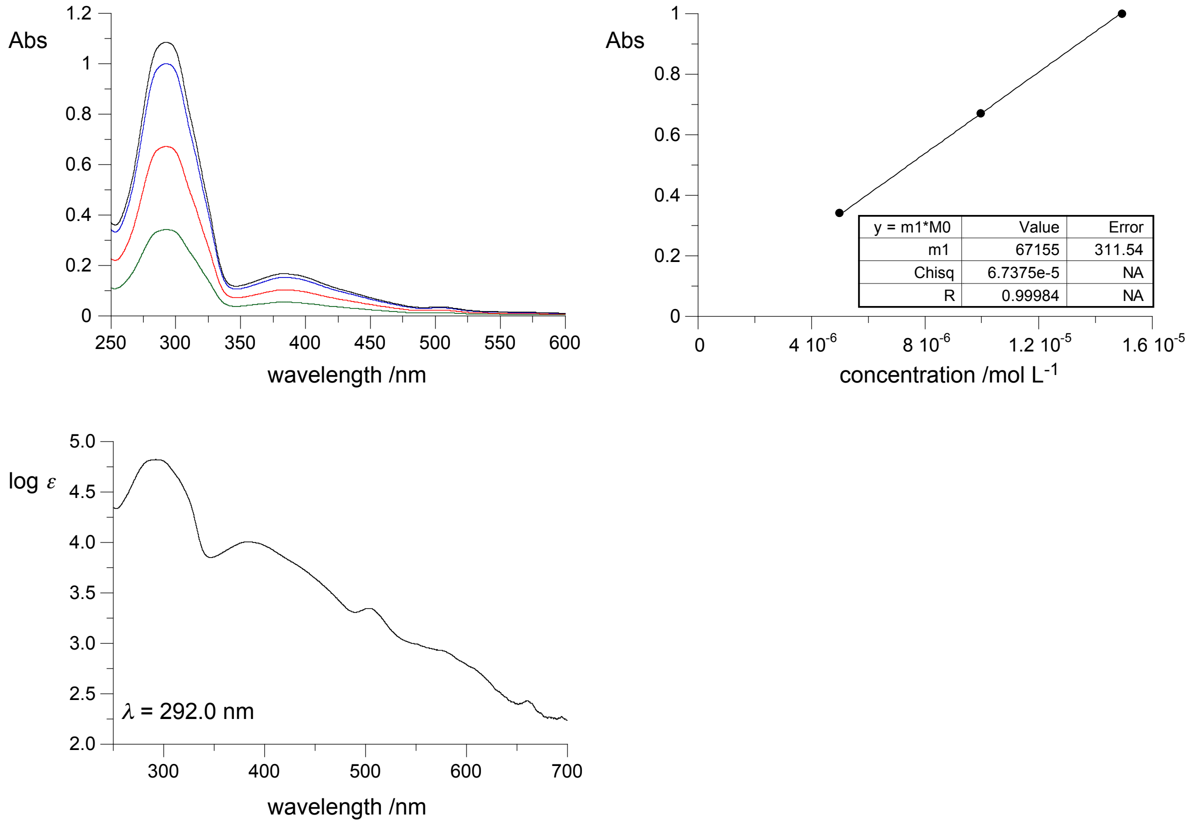

Figure S20. UV-vis absorption spectra of for **Id** recorded in CH_2Cl_2 at four different concentrations (top left), determination of molar extinction coefficient ε at λ = 292.0 nm (top right, best fit function: ε = 67155×conc, r^2 = 0.9998) and molar extinction log (ε) plot (bottom).

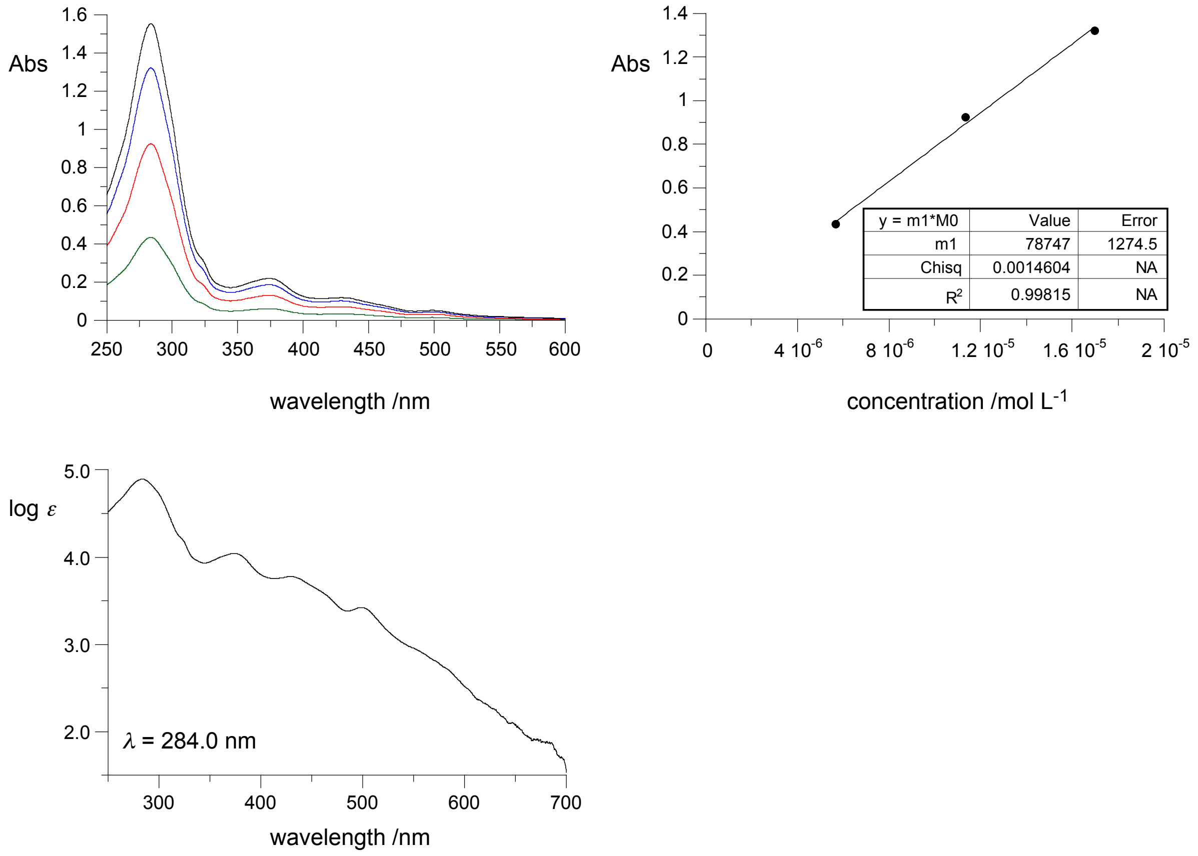

Figure S21. UV-vis absorption spectra of for **IId** recorded in CH_2Cl_2 at four different concentrations (top left), determination of molar extinction coefficient ε at λ = 284.0 nm (top right, best fit function: ε = 78747×conc, r^2 = 0.9982) and molar extinction log (ε) plot (bottom).

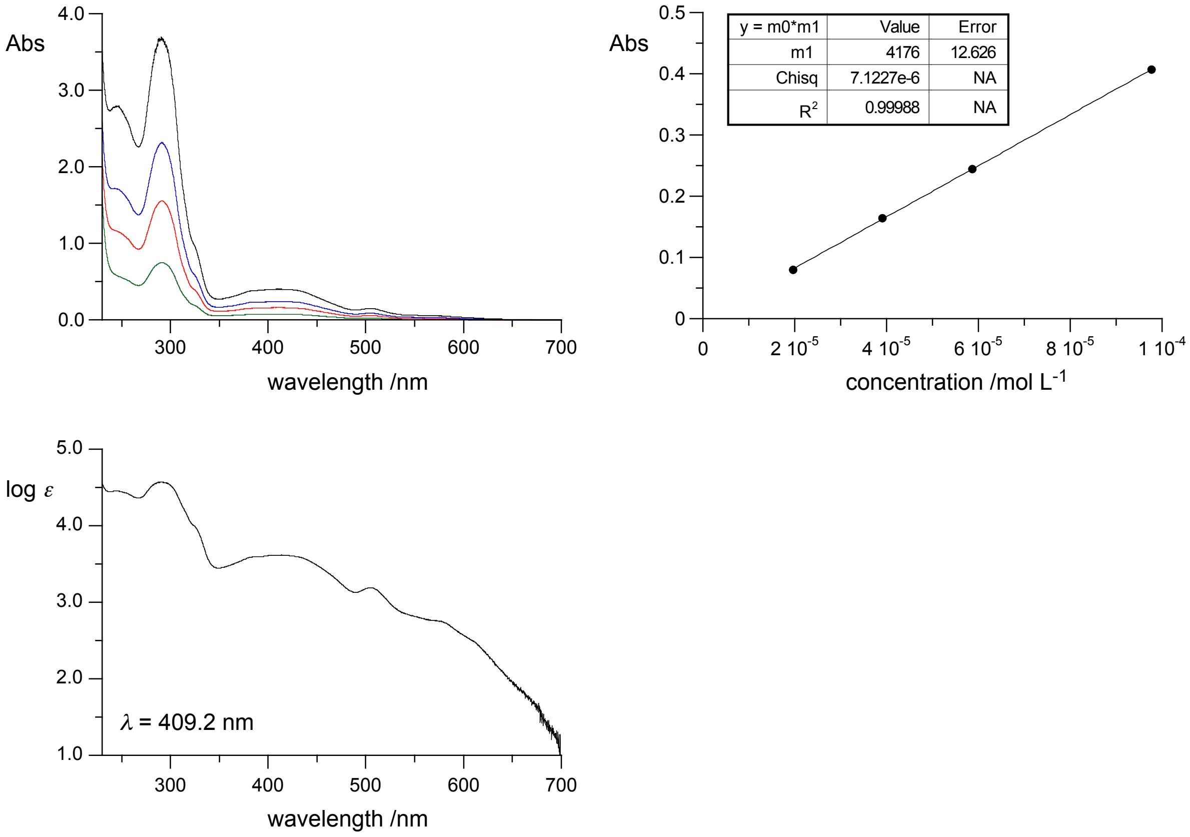

Figure S22. UV-vis absorption spectra of for **Ig** recorded in CH_2Cl_2 at four different concentrations (top left), determination of molar extinction coefficient ε at $\lambda = 409.2$ nm (top right, best fit function: $\varepsilon = 4176\times\text{conc}$, $r^2 = 0.9999$) and molar extinction log (ε) plot (bottom).

3. EPR Spectroscopy

EPR spectra of radicals **I–III** were recorded on an X-band EMX-Nano EPR spectrometer at ambient temperature using dilute and degassed solutions in distilled benzene in a concentration range of $2–5\times10^{-4}$ M. The microwave power was set with the Power Sweep program below the saturation of the signal, modulation frequency of 100 kHz, modulation amplitude of 0.5 G_{pp} and spectral width of 100 G.

Simulation of the spectra was performed with EasySpin (Matlab) using all EPR-active nuclei and DFT results as the starting point including all nitrogen atoms, fluorine atoms and up to 8 hydrogen atoms. The chemically equivalent nuclei, H in the Ph substituent and F in the CF_3, were treated as a group of 2 and 3 identical nuclei, respectively. The resulting *hfcc* values were perturbed several times until a global minimum for the fit was achieved.

The experimental and simulated spectra are shown in Figures S23–S30.

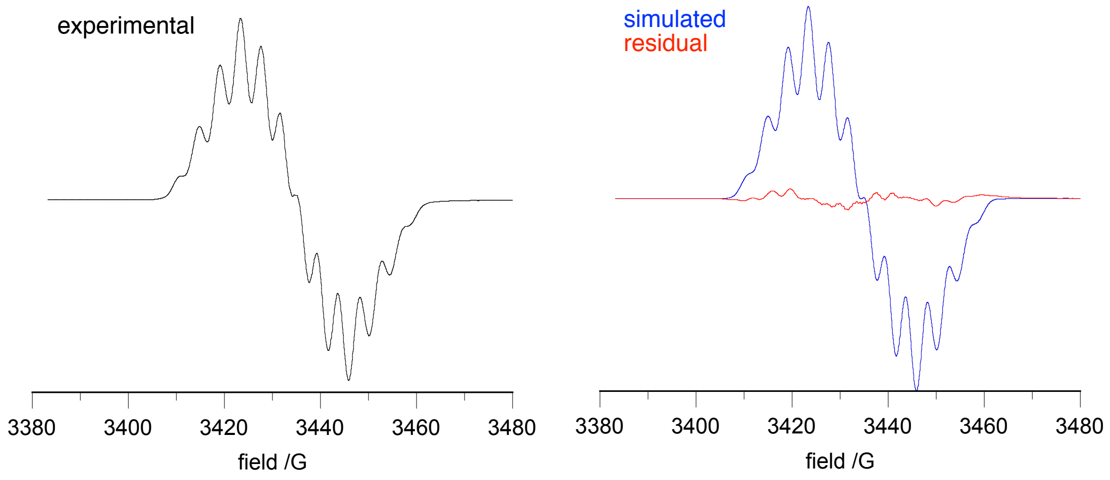

Figure S23. Experimental (black, left), simulated (blue, right) and difference (red, right) spectra for **Ia** recorded in benzene at *ca* 20 °C.

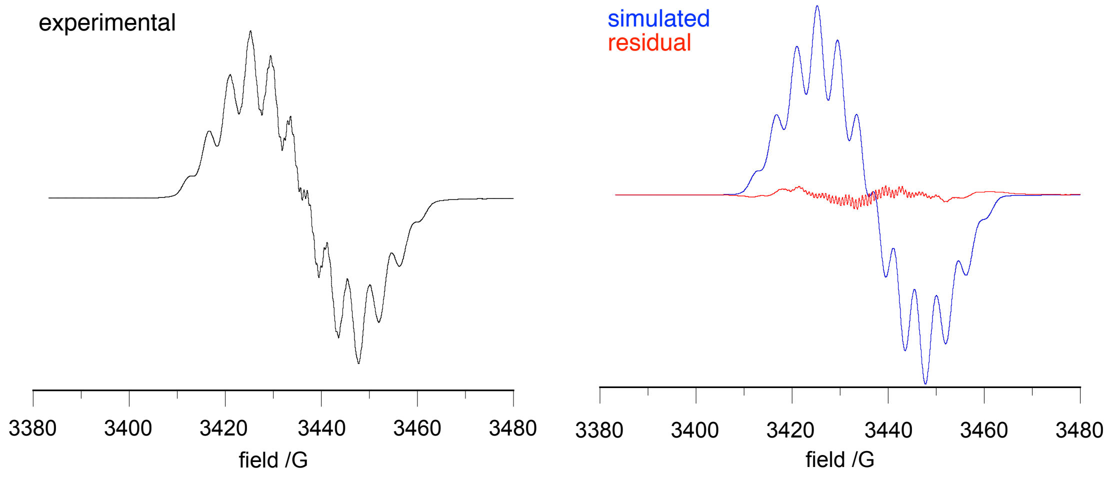

Figure S24. Experimental (black, left), simulated (blue, right) and difference (red, right) spectra for **Ic** recorded in benzene at *ca* 20 °C.

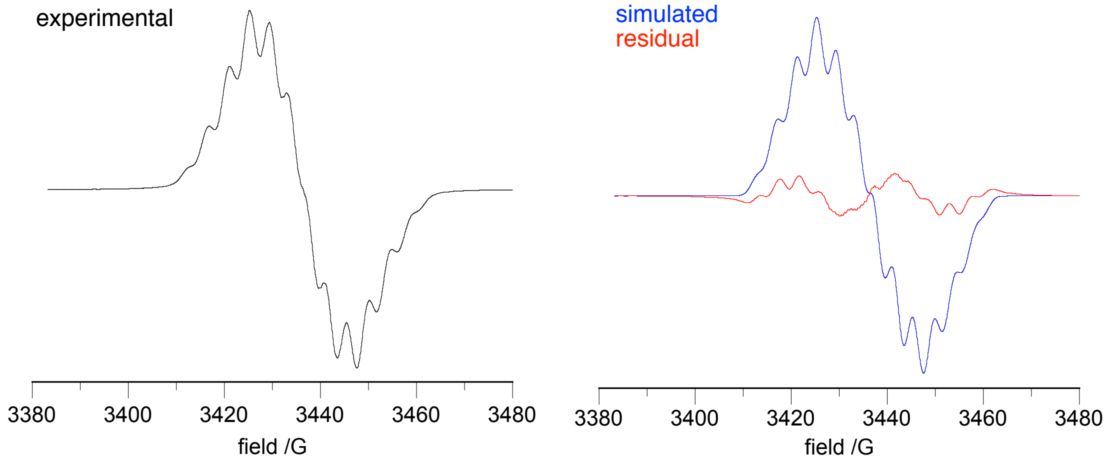

Figure S25. Experimental (black, left), simulated (blue, right) and difference (red, right) spectra for **Id** recorded in benzene at *ca* 20 °C.

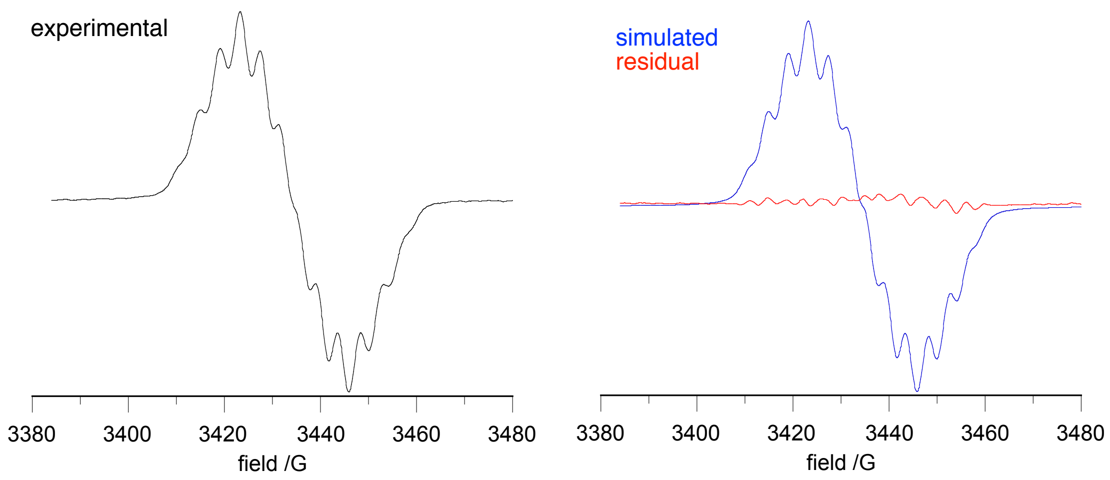

Figure S26. Experimental (black, left), simulated (blue, right) and difference (red, right) spectra for **Ie** recorded in benzene at *ca* 20 °C.

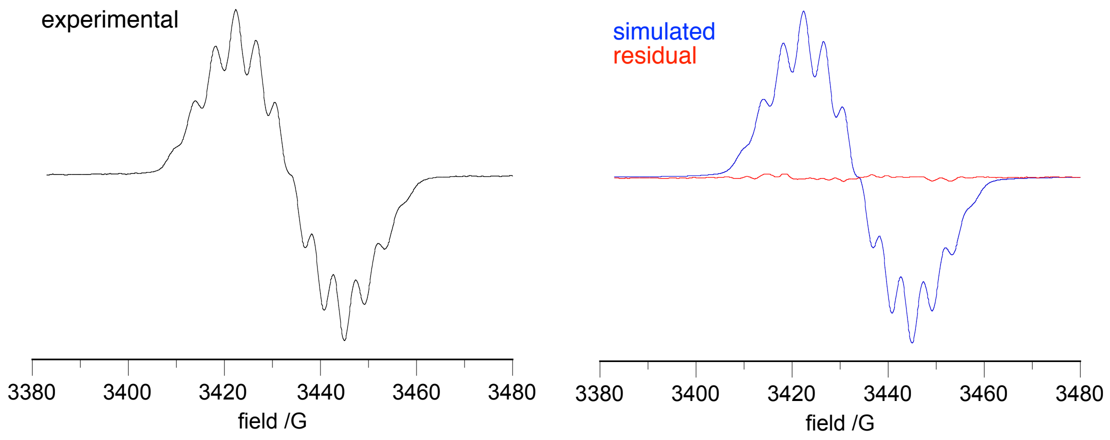

Figure S27. Experimental (black, left), simulated (blue, right) and difference (red, right) spectra for **Ig** recorded in benzene at *ca* 20 °C.

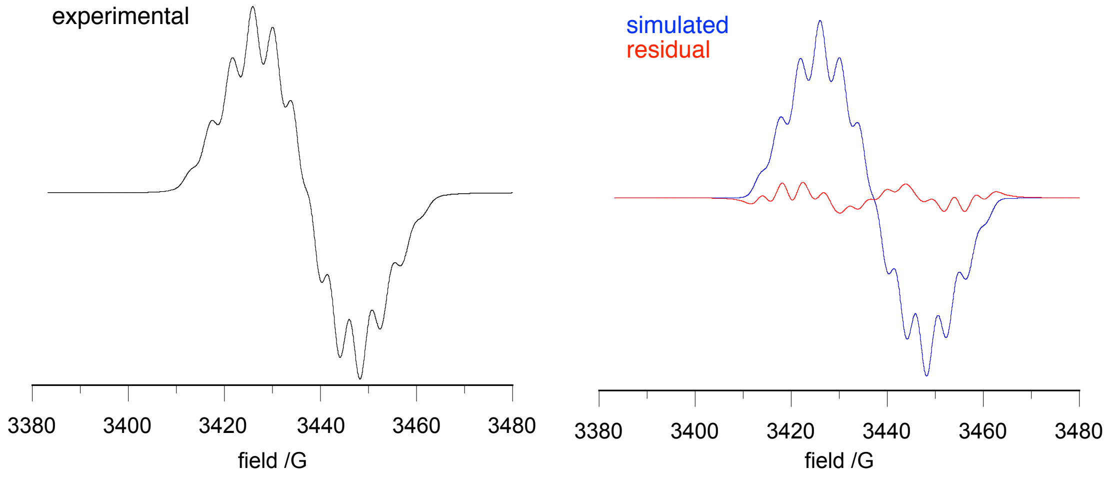

Figure S28. Experimental (black, left), simulated (blue, right) and difference (red, right) spectra for **IIa** recorded in benzene at *ca* 20 °C.

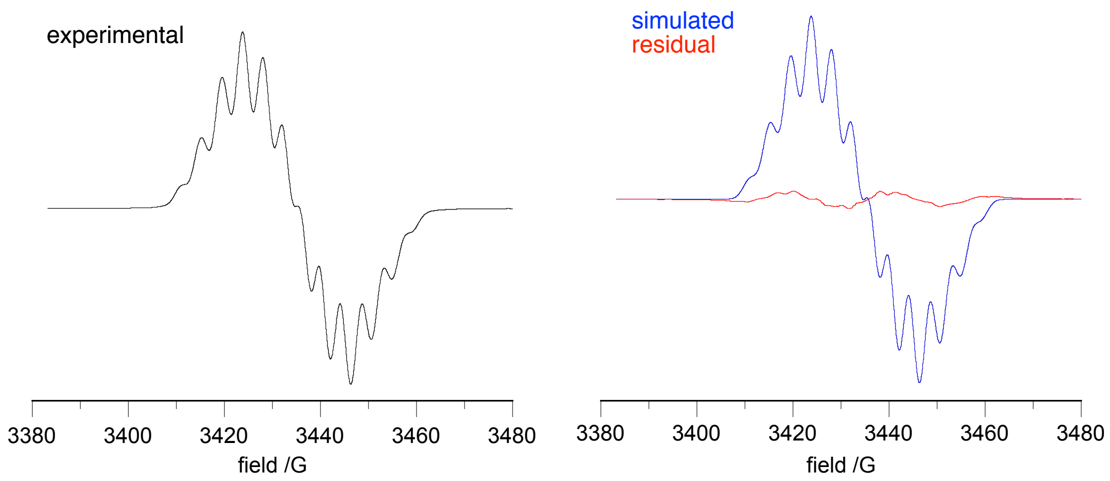

Figure S29. Experimental (black, left), simulated (blue, right) and difference (red, right) spectra for **IIc** recorded in benzene at *ca* 20 °C.

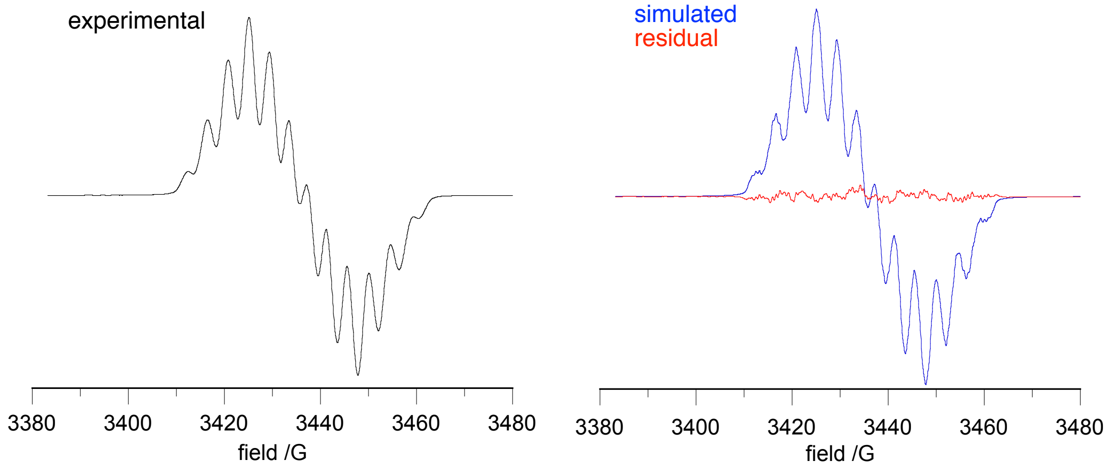

Figure S30. Experimental (black, left), simulated (blue, right) and difference (red, right) spectra for **IIIa** recorded in benzene at *ca* 20 °C.
